# A restricted period for formation of outer subventricular zone defined by Cdh1 and Trnp1 levels

**DOI:** 10.1038/ncomms11812

**Published:** 2016-06-06

**Authors:** Maria Ángeles Martínez-Martínez, Camino De Juan Romero, Virginia Fernández, Adrián Cárdenas, Magdalena Götz, Víctor Borrell

**Affiliations:** 1 Instituto de Neurociencias, Consejo Superior de Investigaciones Científicas and Universidad Miguel Hernández, Sant Joan d'Alacant 03550, Spain; 2 Helmholtz Center Munich, German Research Center for Environmental Health, Institute for Stem Cell Research, 85764 Neuherberg, Germany; 3 Physiological Genomics, University of Munich, 80336 Munich, Germany; 4 SYNERGY, Excellence Cluster of Systems, Neurology, Biomedical Center, 82152 Martinsried-Planegg, Germany

## Abstract

The outer subventricular zone (OSVZ) is a germinal layer playing key roles in the development of the neocortex, with particular relevance in gyrencephalic species such as human and ferret, where it contains abundant basal radial glia cells (bRGCs) that promote cortical expansion. Here we identify a brief period in ferret embryonic development when apical RGCs generate a burst of bRGCs that become founders of the OSVZ. After this period, bRGCs in the OSVZ proliferate and self-renew exclusively locally, thereby forming a self-sustained lineage independent from the other germinal layers. The time window for the brief period of OSVZ bRGC production is delineated by the coincident downregulation of *Cdh1* and *Trnp1*, and their upregulation reduces bRGC production and prevents OSVZ seeding. This mechanism in cortical development may have key relevance in brain evolution and disease.

## Abstract (web summary)

The outer subventricular zone (OSVZ) contains basal radial glial cells (bRGC) involved in cortical expansion in gyrencephalic mammals. Here the authors identify a developmental time window with marked production of bRGCs required to found the OSVZ that is dependent on coincident downregulation of Cdh1 and Trnp1.

## Graphical abstract

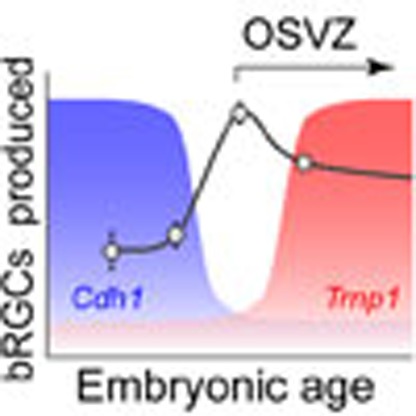

A hallmark of neocortical development in gyrencephalic mammals is the formation of the outer subventricular zone (OSVZ)^1^. This is a specialized germinal layer that emerges at mid-corticogenesis, contains vast numbers of progenitor cells and is crucial for the evolutionary expansion of the mammalian cerebral cortex in neuron number and surface area, thus promoting the emergence of gyrencephaly^1^,^2^,^3^,^4^,^5^,^6^,^7^. To date, the mechanisms regulating the developmental emergence of the OSVZ remain largely unknown.

The OSVZ contains a variety of basal progenitor cells, namely, basal radial glial cells (bRGCs) and intermediate progenitor cells (IPCs)^4^,^5^,^7^,^8^,^9^,^10^. IPCs are multipolar cells expressing the t-box transcription factor Tbr2, and are a major source of neocortical neurons in rodents^11^,^12^,^13^,^14^,^15^,^16^. Basal RGCs resemble the apical RGCs (aRGCs) of the ventricular zone (VZ), including a radially extended basal process and expression of Pax6, but lack attachment to the apical adherens junction (AJ) belt^4^,^8^,^9^,^10^,^17^,^18^,^19^. Basal RGCs are scarce in lissencephalic species^17^,^18^, but very abundant in gyrencephalic mammals, where they are highly neurogenic and accumulate prominently in the OSVZ^4^,^8^,^9^,^10^,^20^. Although lissencephalic mammals may develop a vestigial form of an OSVZ, it is in gyrencephalic species where this is largest, most complex and mitotically active^1^,^4^,^8^,^10^,^21^,^22^,^23^.

In mouse, video microscopy studies of brain slices *in vitro* have revealed that IPCs and bRGCs have very poor self-renewing capacity, and their pool can only be maintained by the continuous production from aRGCs in the VZ^13^,^14^,^15^,^16^,^18^,^24^. This process is finely regulated by the action of Trnp1, a DNA-binding protein that limits IPC and bRGC production^7^,^25^,^26^. Similar *in vitro* analyses have shown that this process is much more complex in gyrencephalic species such as ferret, macaque and human, where IPCs and bRGCs in the OSVZ have been reported to proliferate and self-renew to some extent locally^4^,^8^,^10^,^27^. However, it is not known when and where these cells first arise and if feeding into these progenitor pools continues throughout development.

Here we present the first *in vivo* analysis of progenitor cell lineage dynamics in ferret, a gyrencephalic carnivore, performed at multiple developmental stages and providing us with unprecedented insights into OSVZ formation and expansion in the intact embryo. Although phylogenetically distant from humans, cortical development in ferret shares many key features with humans and other primates, and uniquely allows *in vivo* and *in utero* manipulations. We find that the OSVZ is initiated during a brief period of embryonic development, when aRGCs undergo self-consuming divisions to massively produce bRGCs, which migrate past the inner subventricular zone (ISVZ) and become the founder cells of the OSVZ. After closure of this restricted period, aRGCs in VZ continue generating bRGCs, but only for the ISVZ, while progenitor cells in the OSVZ follow a completely independent lineage. The timing and duration of this restricted period depends on the dynamic regulation of *Cdh1* and *Trnp1* expression levels, when low expression of both genes is necessary to open this period, and high levels are sufficient to impair bRGC generation. Genetic abrogation of this restricted period reduces markedly seeding of bRGCs to the OSVZ and their abundance for the remaining cortical development, suggesting that its occurrence and modulation may have played an essential role in the evolutionary emergence and expansion of the OSVZ.

## Results

### Late OSVZ progenitor cells follow an independent lineage

**Figure 1:**
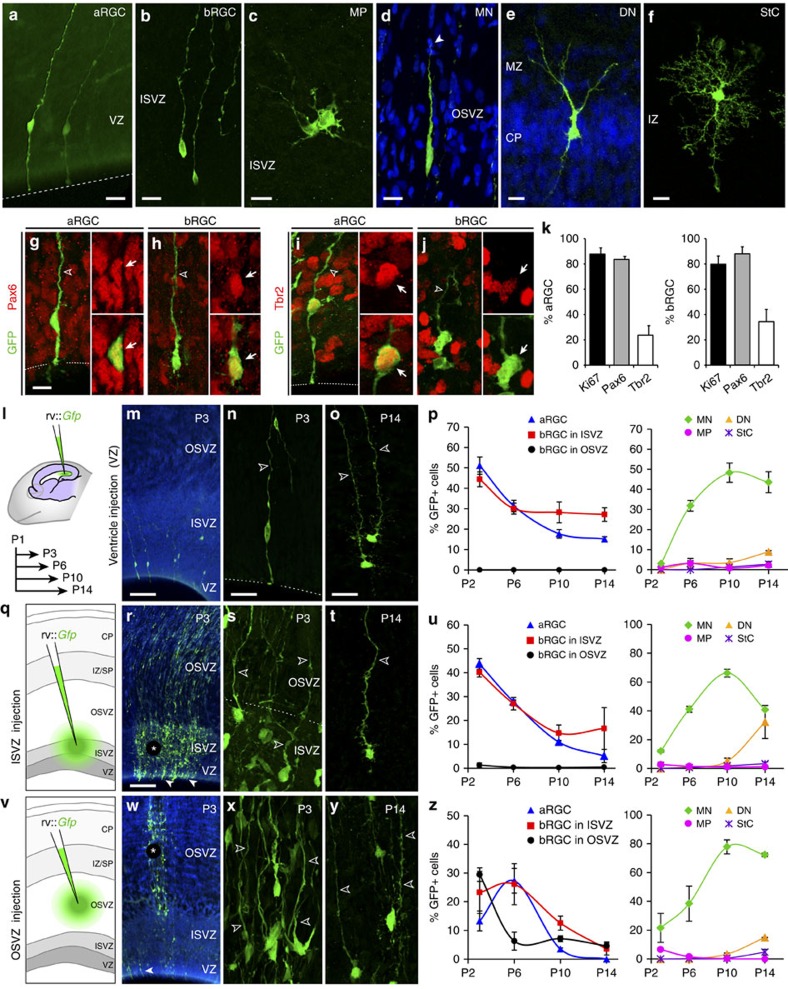
Postnatal VZ and ISVZ do not generate bRGCs for the OSVZ. (**a**–**f**) Examples of GFP+ cells after injection of rv::*Gfp* into VZ (lateral ventricle), ISVZ or OSVZ at P1, with stereotyped morphologies: apical radial glia cells (aRGCs), basal radial glia cells (bRGCs), multipolar cells (MP), migrating neuron (MN), differentiating neuron (DN) and star-like cells (StCs). DNs typically had the cell soma in the cortical plate (CP) and a single apical dendrite branching in the marginal zone (MZ). IZ, intermediate zone. (**g**–**k**) GFP+ aRGCs in VZ (**g**,**i**) and bRGCs in ISVZ (**h**,**j**) at P6 after ventricular injection of rv::*Gfp* at P1, showing expression of Pax6 (**g**,**h**) and Tbr2 (**i**,**j**) in both populations. (**k**) Abundance of aRGCs and bRGCs expressing Ki67, Pax6 or Tbr2 (aRGCs, *n*=57 cells, Ki67; 82 cells, Pax6; 61 cells, Tbr2; 3 animals; and bRGCs, *n*=72 cells, Ki67; 59 cells, Pax6; 31 cells, Tbr2; 3 animals). (**l**–**u**) P1 ferrets were injected with rv::*Gfp* into the lateral telencephalic ventricle to infect VZ (**l**–**p**) or injected locally into ISVZ (**q**–**u**), and analysed at various subsequent stages (**p**,**u**). Data refer to GFP+ cells across the cortical thickness. Cell lineages from these layers contained aRGCs in VZ (**n**) and abundant bRGCs in ISVZ throughout development (**o**,**t**; open arrowheads indicate the basal process), but null presence in OSVZ (*n*=962–5,544 cells per group; Supplementary Table 1). (**v**–**z**) Ferrets were injected with rv::*Gfp* locally into OSVZ at P1 and analysed at later stages (**z**). GFP+ bRGCs were abundant in OSVZ at all survival times (**x**,**y**), demonstrating local bRGC production (*n*=420–3,742 cells per group; Supplementary Table 1). The relative abundance of GFP+ aRGCs and bRGCs decreased rapidly from P3 to P6 regardless of the injection site, due to the progressive accumulation of GFP+ migrating neurons (MNs; **p**,**u**,**z**). Images in **r** and **w** show clusters of GFP+ cells at the injection site (asterisks), and cells retrogradely labelled in VZ (solid arrowheads). Values are mean±s.e.m. in **k**,**p**,**u** and **z**. Scale bars, 20 μm (**a**–**f**); 10 μm (**g**–**j**); 100 μm (**m**–**y**) low magnifications, 20 μm details.

To define the germinal layers generating OSVZ progenitor cells *in vivo*, we began by tracing the lineage of cortical progenitors. We injected *Gfp*-encoding retroviral vectors (rv::*Gfp*) into postnatal day 1 (P1) ferrets, stage equivalent to mouse embryonic day (E) 15, rat E16 and 16 gestational weeks in human cerebral cortex, and analysed the lineage progression of infected progenitor cells at subsequent stages. We chose P1 because this is when OSVZ proliferation and bRGC abundance are maximal in this species^4^,^20^,^28^. The lineage of VZ progenitors was selectively labelled by injecting rv::*Gfp* into the lateral telencephalic ventricle, thereby only transducing progenitor cells in contact with the ventricular surface, whereas the lineage of progenitors in ISVZ and OSVZ was labelled by local rv::*Gfp* injections into these layers (see Methods). Multiple cell populations were labelled across cortical layers regardless of the injection site, including cells with typical morphology of aRGCs, bRGCs, multipolar cells resembling IPCs (MP), bipolar cells resembling migrating neurons, differentiating neurons (DNs) and cells with star-like glial morphology (StC), which included cells in the astrocyte and oligodendrocyte lineages (Fig. 1a–f; Supplementary Figs 1 and 2). Analyses of marker expression with morphology confirmed the identity of aRGCs and bRGCs by their expression of Ki67 and Pax6, and also showed that 24–34% of them expressed the T-box transcription factor Tbr2, as in primates^8^ (Fig. 1g–k).

**Figure 2:**
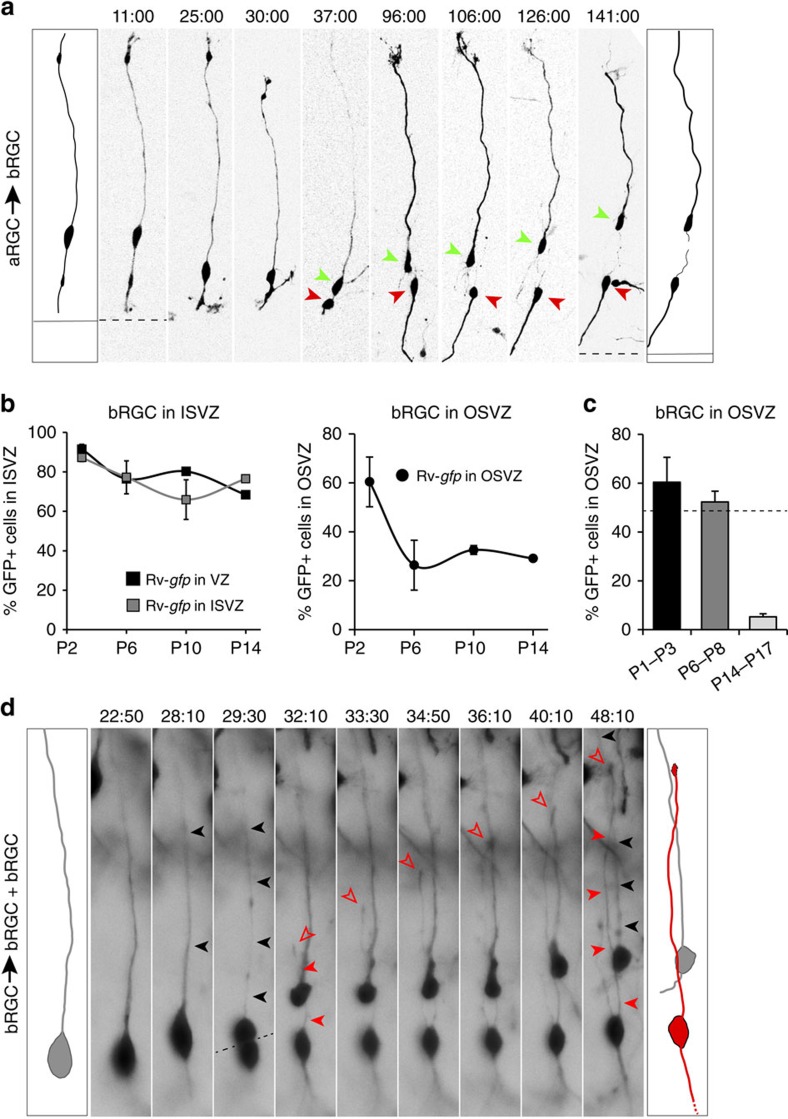
Postnatal aRGCs generate ISVZ bRGCs, while OSVZ bRGCs are generated by self-amplification. (**a**) Time-lapse imaging frames of an aRGC dividing apically, between 30 and 37 h of imaging time, to generate a bRGC (green arrowhead) retaining the basal process. The apical cell (red arrowhead) retained the apical process. (**b**) Abundance of GFP+ bRGCs located in ISVZ after VZ and ISVZ infections at P1 or in OSVZ after OSVZ injections at P1, and analysed at the indicated ages (mean values+s.e.m.; *n*=420–5,544 cells, 2–5 animals per group; Supplementary Table 1). (**c**) Abundance of GFP+ bRGCs in OSVZ at the indicated postnatal stages after injections of rv::*Gfp* in OSVZ and 2–3 days of survival. Data are mean±s.e.m. (*n*=420 cells, 3 animals, P1–P3; 1,288 cells, 4 animals, P6–P8; 819 cells, 2 animals, P14–P17); dashed line indicates 50%. (**d**) Time-lapse imaging frames of a bRGC in OSVZ with a basal process (black arrowheads), undergoing a near-horizontal division at *t*=29:30 hours (dashed line) to generate two bRGCs. The basal daughter cell (top) retained the maternal basal process, whereas the apical daughter (bottom) grew a new basal process (solid red arrowheads) tipped with a small growth cone (red open arrowheads).

On infection of VZ progenitors with rv::*Gfp* at P1, by P3 we found that 50.6% of GFP+ cells were aRGCs and 45.3% bRGCs (Fig. 1l–n,p). The production of bRGCs from aRGCs was confirmed by two-photon video microscopy in slice cultures (Fig. 2a), in agreement with previous reports^18^,^26^,^27^,^29^. Remarkably, the cell bodies of all bRGCs were found in ISVZ (hereon referred to as bRGCs in ISVZ) and none in OSVZ (hereon referred to as bRGCs in OSVZ; Fig. 1p). Because the OSVZ is further away from the VZ than ISVZ, we reasoned that 2 days of survival might be insufficient time for newly generated bRGCs to migrate from VZ to OSVZ. To test this, we next allowed longer survival periods after rv::*Gfp* injection. GFP labelling was traced for up to 2 weeks post injection, but the cell bodies of bRGCs continued absent from the OSVZ while very abundant in ISVZ (Figs 1o,p and 2b). These results provided the first demonstration *in vivo* that aRGCs are an abundant source of bRGCs in gyrencephalic species, but also that at late stages of cortical development these are solely destined to the ISVZ, without contributing to the OSVZ.

Next, we reasoned that if bRGCs in OSVZ were not produced directly from VZ, they might be generated indirectly via bRGCs in ISVZ. The above results seemed to rule out this possibility, as GFP+ bRGCs were not seen in OSVZ even long after they had been observed in ISVZ (Fig. 1o,p). An alternative possibility was that bRGCs in OSVZ were produced by other types of progenitor cells resident in ISVZ and/or not derived from the postnatal VZ. In such case, bRGCs in OSVZ would only be revealed by direct labelling of ISVZ progenitors. To test this, we injected rv::*Gfp* locally into the ISVZ of P1 ferrets (Fig. 1q,r). After 2 days of survival, abundant aRGCs and bRGCs were labelled in VZ and ISVZ, respectively, but were virtually absent in OSVZ (Fig. 1s,u). At longer survival times, bRGCs continued to represent the vast majority of GFP+ cells in ISVZ, but remained absent from OSVZ (Fig. 1t,u; Fig. 2b), demonstrating that bRGCs in OSVZ were not produced by progenitor cells in the postnatal ISVZ. Taken together, our results demonstrated that at peak stages of OSVZ expansion, VZ and ISVZ are abundant sources of bRGCs that will exclusively populate the ISVZ, but never contribute to the OSVZ.

### Abundant generation of bRGCs in the postnatal OSVZ

To elucidate if bRGCs in the postnatal OSVZ were generated locally, we injected rv::*Gfp* into the OSVZ of P1 ferret kits (Fig. 1v,w). At P3, we found a majority of GFP+ cells in the OSVZ displaying bRGC morphology (Figs 1x,z and 2b), a proportion that decreased by P6 to then remain similar until P14 (Figs 1y,z and 2b), demonstrating that OSVZ bRGCs are abundantly generated in the postnatal OSVZ. To investigate the dynamics of this bRGC production in OSVZ, we injected rv::*Gfp* at various stages and analysed after only 2 days of survival in each case. At P3 and P8, bRGCs represented 60–52% of cells born in OSVZ (Fig. 2c), indicating their net increase (see Methods). Video microscopy in slice cultures demonstrated self-amplification of bRGCs, including *de novo* generation of a basal process (Fig. 2d) as in primates^8^,^10^. Although OSVZ injections also labelled cells in ISVZ and VZ via their radial process (see Methods and Fig. 1w), our above analyses showed that these layers never generate OSVZ bRGCs, and therefore these were being generated only locally by rv::*Gfp*-infected progenitors within the postnatal OSVZ.

At all these stages, IPCs (defined as the subset of MP cells that are Ki67+, Tbr2+) in ISVZ and OSVZ were a very small minority of cortical progenitors, as opposed to the mouse SVZ (Fig. 1p,u,z; Supplementary Fig. 3; Supplementary Note 1). Moreover, the few IPCs in OSVZ were not generated by VZ or ISVZ progenitors, but mainly locally within OSVZ similar to bRGCs (Supplementary Fig. 2). Taken together, our findings demonstrated that the OSVZ is a unique niche of progenitor cell production at postnatal stages, following a lineage independent from progenitors in VZ and ISVZ, and where the vast majority of progenitors are bRGCs that expand by self-amplification.

### Embryonic initiation of OSVZ by founder bRGCs seeded from VZ

**Figure 3:**
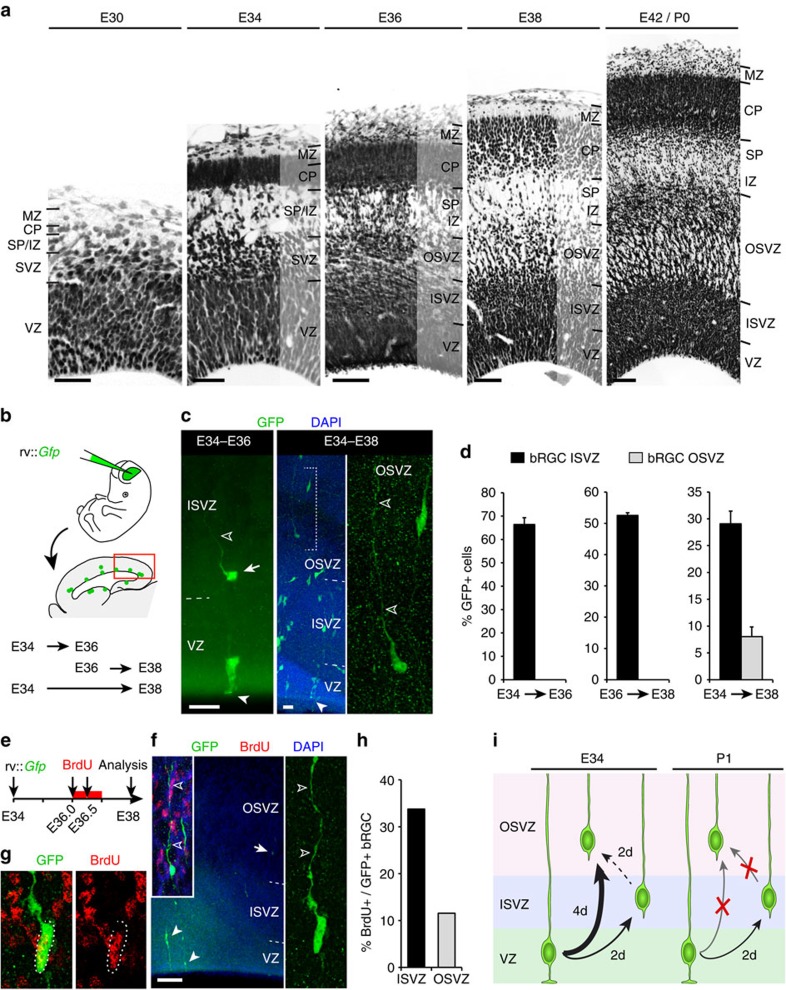
Embryonic aRGCs generate directly bRGCs for the OSVZ. (**a**) Nissl stains of the ferret embryonic neocortex illustrate the developmental progression of germinal layers and the first histological recognition of OSVZ between E34 and E36. (**b**–**d**) Ferret embryos received a ventricular injection of rv::*Gfp* at E34 or E36, and developed *in utero* until E36 or E38. GFP+ bRGCs were found in OSVZ after 4 days of survival (E34–E38, detail), but only in ISVZ after 2 days (E34–36, arrow; *n*=931 cells, 8 embryos; E36–38, *n*=104 cells, 2 embryos; E34–38, *n*=1,830 cells, 7 embryos; mean values+s.e.m.). (**e**–**h**) VZ progenitors were labelled with rv::*Gfp* at E34, BrdU was administered 2 days later and were analysed at E38 (**f**,**g**). Arrow in **f** points at the bRGC magnified in inset. Very few GFP+ bRGCs in OSVZ contained BrdU (**f**, right; **f**,**g**; *n*=229 cells, 5 embryos), indicating that most were directly generated from VZ. In **c** and **f**, solid arrowheads indicate aRGCs and open arrowheads indicate basal process of bRGCs. (**i**) At E34, aRGCs generate bRGCs that reach the ISVZ 2 days later (E36) and OSVZ 4 days later (E38). Few bRGCs in OSVZ are generated between E36 and E38 (likely from ISVZ), the majority being generated between E34 and E36 directly from VZ. Postnatally, aRGCs generate bRGCs for the ISVZ, but neither generates bRGCs for the OSVZ. Scale bar, 30 μm (E30 and E34; **a**), 100 μm (E36; **a**), 150 μm (E38; **a**), 200 μm (E42/P0, **a**); 25 μm (**c**); 150 μm (**f**).

In spite of our above findings, the key question of where and when OSVZ progenitors (mainly bRGCs) arise initially remained open. Given that all cortical cells derive from the early embryonic VZ at some point^30^, we focused on earlier stages to determine the origin of OSVZ bRGCs. In ferret, the OSVZ is first distinguishable at E36 (equivalent to mouse E13), 6 days before birth (E42/P0; Fig. 3a), so we traced VZ progenitors with injections of rv::*Gfp* into the lateral telencephalic ventricle at E34 (equivalent to mouse E12.5) and E36 *in utero* (Fig. 3b). Two days after infection, numerous bRGCs expressed GFP in ISVZ, but none in OSVZ (Fig. 3c,d). Because 2 days of survival might be insufficient time for new bRGCs to migrate from VZ to OSVZ, we repeated the injections at E34, but now analysing at E38. Four gestational days after infection (E34–E38), 21.7% of bRGCs were in OSVZ (representing 8.0±1.8% of all GFP+ cells; Fig. 3d). These results demonstrated that bRGCs in the postnatal OSVZ had been originally produced by aRGCs at embryonic stages, though not anymore at postnatal stages.

The lineage tracing between E34 and E38 potentially allowed for several rounds of cell division, so we could not distinguish if bRGCs in OSVZ had been generated in a single step directly by aRGCs in VZ at E34 or indirectly following a second round of division in ISVZ between E36 and E38. To distinguish between these possibilities, we injected rv::*Gfp* at E34, administered BrdU at E36.0 and E36.5 to label cells cycling between E36 and E37 (S-phase ≈12 h)^20^,^31^, and analysed at E38 (Fig. 3e). BrdU administration was interrupted at E37 to avoid labelling bRGCs that might have reached the OSVZ early. Only 11.5% of all bRGCs in OSVZ contained BrdU (Fig. 3f–h), indicating that the majority had been directly generated by aRGCs in VZ before E36. In addition, rv::*Gfp* infection of VZ at E34 labelled some IPCs in OSVZ at E38 (MP, Ki67+; 0.46±0.37% of GFP+ cells), unlike their null labelling at postnatal stages (Supplementary Fig. 2). Hence, aRGCs in the embryonic VZ produce bRGCs and IPCs that migrate through the ISVZ *en route* to the OSVZ, a process not occurring after birth (E42) in the ferret cerebral cortex (Fig. 3i). Together, our results demonstrated that the OSVZ develops into two distinct phases: an early period of progenitor cell seeding from the VZ and a later period of self-amplification independent from other germinal layers.

### A restricted period to massively produce OSVZ bRGCs from VZ

**Figure 4:**
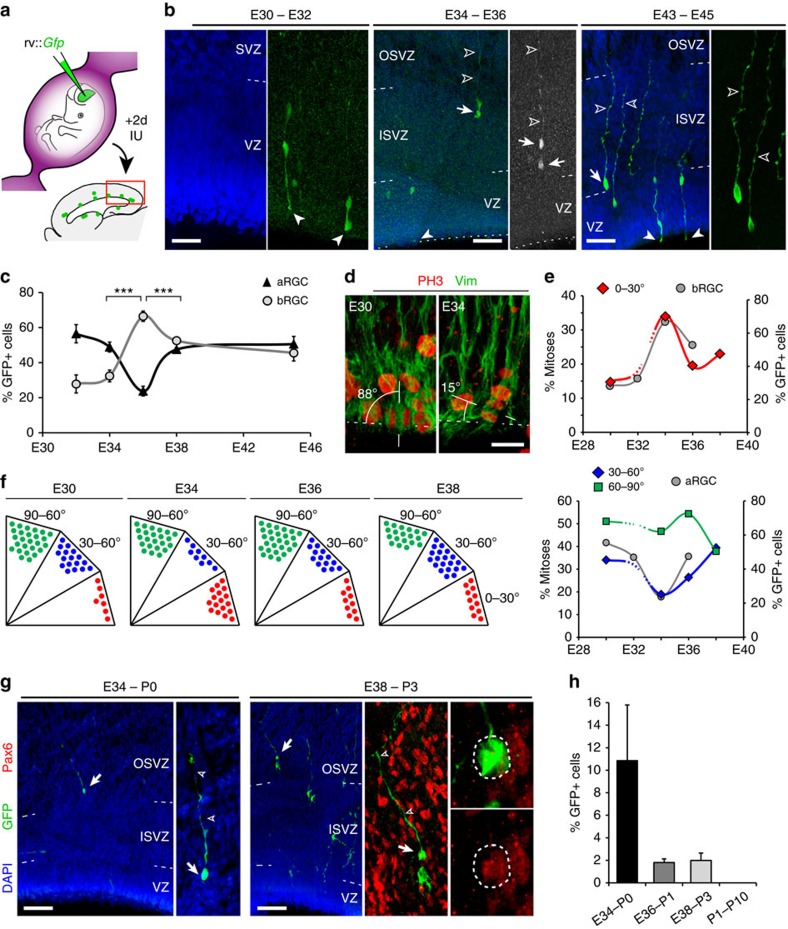
Transient peak of bRGC production from aRGCs at the onset of OSVZ. (**a**–**c**) Tracing of bRGC production from aRGCs along development. Ferret embryos aged E30, E32, E34, E36 and E43/P1 kits received an intraventricular injection of rv::*Gfp* and were analysed 2 days later. (**b**) GFP labelling in embryos injected as indicated; solid arrowheads indicate ventricular end feet of aRGCs, arrows indicate bRGCs and open arrowheads indicate basal process. (**c**) Abundance of GFP+ aRGCs and bRGCs at the indicated ages. Production of bRGCs peaked at E36, when aRGCs self-consumed (<50%); ***P<0.001, *χ*^2^-test; *n*=170 cells, E32; 304 cells, E34; 931 cells, E36; 104 cells, E38; 1,094 cells, E45/P3; 2–8 embryos per group; mean values±s.e.m. (**d**–**f**) Analysis of cleavage orientation plane of VZ mitoses with respect to ventricular surface (dashed lines; *n*=91 cells, E30; 90 cells, E34; 124 cells, E36; 69 cells, E38; 2–8 embryos per group). Each dot in **f** represents 2% of mitoses. Data in **e** show that developmental variations in horizontal (0–30^o^, red) and oblique (30–60°, blue) cleavage planes were paralleled by bRGC and aRGC production, respectively, after rv::*Gfp* infection of VZ at those ages (grey curves). (**g**,**h**) Long-term GFP labelling of bRGCs in OSVZ (arrows) after intraventricular injection of rv::*Gfp* and analysis at the indicated ages. High magnifications show details of these bRGCs in OSVZ exhibiting a long basal process (open arrowheads) and expressing Pax6 (single confocal plane). (**h**) Abundance of GFP+ cells corresponding to bRGCs in OSVZ at the indicated ages (E34–P0, *n*=513 cells, 2 kits; E36–P1, *n*=958 cells, 3 kits; E38–P3, *n*=906 cells, 3 kits; P1–P10, *n*=1,499 cells, 5 kits; mean values+s.e.m.). Note that embryonic VZ progenitors generate bRGCs that populate the postnatal OSVZ, in contrast to postnatal VZ progenitors that do not. Scale bar, 40 μm (E32; **b**), 75 μm (E36 and E45; **b**); 15 μm (**d**); 100 μm (**g**).

To precisely define the embryonic period during which the VZ produces bRGCs (for any germinal layer), we monitored the developmental dynamics of bRGC generation from aRGCs with rv::*Gfp* injections *in utero* and 2-gestational day survivals (Fig. 4a). At all ages, virtually all cells born from VZ (GFP+) were aRGCs or bRGCs (Fig. 4b,c), but their proportions changed significantly along development. At E32 and E34, 47–56% of GFP+ cells were aRGCs, and only 29–34% bRGCs. Between E34 and E36, the production of bRGCs increased markedly to represent 66% of GFP+ cells at E36, paralleled by the self-consumption of aRGCs (24%; aRGCs are not regenerated on division, but produce two daughter cells different from the mother aRGC; see Methods; Fig. 4b,c). This situation lasted only 2 days, and by E38 and P1, the generation of bRGCs was down to 53–45% of GFP+ cells, respectively, and aRGCs were back up to 47–51% (Fig. 4b,c).

Given the short-survival time of these lineage experiments, and that the originally infected cells were aRGCs, we interpreted changes in the percentage of GFP+ aRGCs as changes in cell-fate decisions at the population level: self-amplification (>50% of GFP+ cells are aRGCs), self-renewal (50%) or consumption (<50% are aRGCs). Cell-fate decisions by aRGCs co-vary with their angle of mitotic cleavage plane, where vertical cleavage planes (60–90° to the VZ surface) occur in symmetric self-amplifying divisions and horizontal cleavage planes (0–30° to VZ surface) occur in asymmetric divisions generating bRGCs^17^,^25^,^29^,^32^,^33^. In agreement with this notion, we found that the proportion of horizontal mitotic cleavage planes in VZ peaked transiently at E34, following a temporal dynamics parallel to the depletion of aRGCs and the production of bRGCs, which are maximal for VZ progenitors infected at E34 (Fig. 4d–f; Supplementary Fig. 4). In summary, bRGCs are generated by aRGCs following self-consuming divisions with a burst between E34 and E36.

Unfortunately, these lineage tracings across embryonic development were done with only 2 days of survival, which is too short to determine if the bRGCs generated were destined to OSVZ or not (Fig. 3d). To investigate whether the embryonic production of OSVZ bRGCs from the VZ was narrowly restricted to the burst period between E34 and E36 (Fig. 4c), or it continued at later stages, we infected VZ progenitors *in utero* with rv::*Gfp* at various stages and allowed 7–8 days of survival in each case, providing sufficient time for labelled cells to migrate and reach the OSVZ. We observed most OSVZ bRGCs in pups infected at E34, very few when infections were done at E36 and E38, and none in P1 injections (Fig. 4g,h). These experiments thus allowed us to identify and delineate the period of OSVZ bRGC production/seeding from the VZ with a prominent peak at E34 and very limited contributions at E36 and E38. As no OSVZ bRGCs are generated any longer at P1, their production is strikingly restricted to a rather brief period of cortical development (E34–P0), with a burst or maximum peak between E34 and E36.

### *Cdh1* and *Trnp1* restrict the period for OSVZ generation

**Figure 5:**
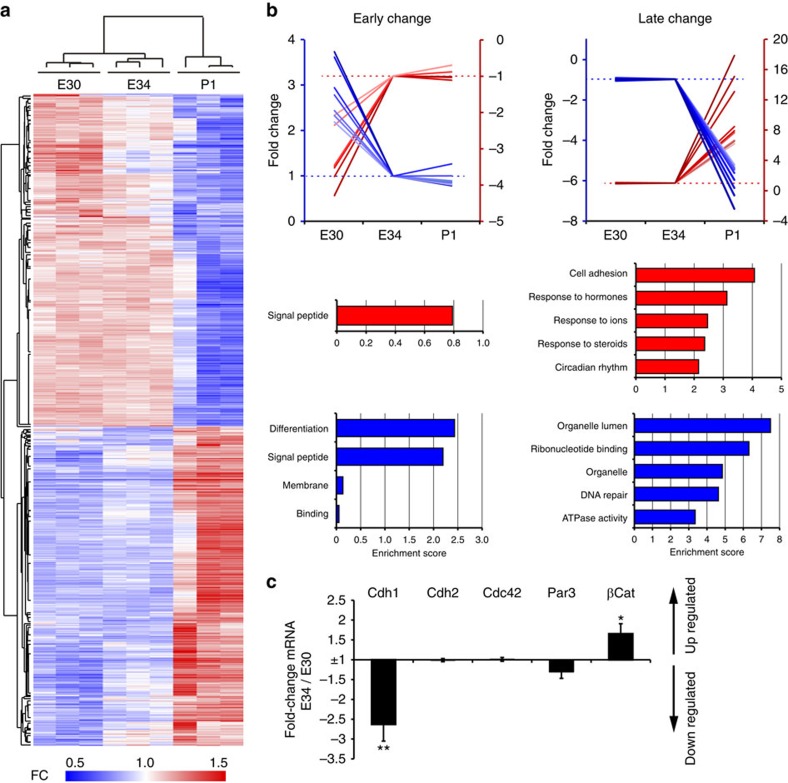
Differential gene expression in VZ across developmental stages. (**a**) Heatmap of unsupervised hierarchical clustering of gene probes differentially expressed in VZ between E30, E34 and P1 (FC, fold change), and dendrogram of similarity between samples. Each lane is a biological replica. The vast majority of probes were differentially expressed between E34 and P1, but not between embryonic stages. (**b**) Examples of developmental expression profiles of differentially expressed genes, and for each profile the functional gene annotation terms as analysed and clustered using DAVID. The five clusters with the highest enrichment scores for up- and downregulated genes are shown. The vast majority of DEGs (FC>2, *P*<0.05) had ‘late-change' profile (increase, red axis; decrease, blue axis; only 10 genes with highest FC in each axis are plotted), whereas much fewer had ‘early' change profiles (41 probes). Red axes are for data represented in red, and blue axes for data in blue. (**c**) Quantitative RT–PCR data for candidate gene expression levels in VZ, expressed as FC between E34 and E30 (mean values±s.e.m.). Negative values indicate lower expression at E34 and positive values indicate higher expression at E34. *Cdh1* expression decreased >2.5-fold between these two ages, whereas *βCat* increased 1.6-fold and other apical complex protein genes remained unchanged. *P<0.05, **P<0.01, *t*-test.

To identify candidate genes regulating the dynamics of bRGC production by aRGCs, we searched for differential gene expression in VZ between three key stages: E30, E34 and P1. Using a ferret-specific microarray^34^,^35^, we identified 1,852 differentially expressed genes (DEGs) between at least two stages (false discovery rate <0.05, fold change >2; Fig. 5a). Only 59 DEGs were found between E30 and E34 (27 upregulated and 32 downregulated), whereas 1,822 genes changed between E34 and P1 (871 upregulated and 951 downregulated). Because the production of bRGCs increased in the early period (E30–E34), but then decreased in the late period (E34–P1; Fig. 4c), we discarded DEGs whose expression levels changed in the same direction in both periods (first up- then again upregulated or first down- then again downregulated). Following the same reasoning, from the remaining DEGs we selected those whose expression levels changed in opposite directions between early and late phases (first up- then downregulated or down- then upregulated). Only one gene followed this ‘peak' profile of expression, which encodes a synaptic protein (Sv2b) that is not expressed in mouse cortical progenitors (www.genepaint.org), and has no known function in cell proliferation, cell fate or delamination^36^. At this point, we considered that the early phase (E30–E34) and late phase (E34–P1) might be regulated by different genes, so we distinguished between genes changing only in the early phase (‘early-change' profile) or the late phase (‘late-change' profile; Fig. 5b; Supplementary Tables 2 and 3). Some of the early genes are related to axonal navigation (Slit and EphA5), whereas others are implicated in human diseases (LYPD1 and FAM167A). Late genes also include human disease genes such as BATF2 (acute stress disorder), GJB6 (sensorineural deafness and a variety of skin disorders) or APOE (lipoproteinemias), as well as a variety of biological functions such as cell adhesion (NDNF and THBS1), ribonucleotide binding (NABP1 and MSH3) or interaction with various organelles (FKBP4 and MARS2).

**Figure 6:**
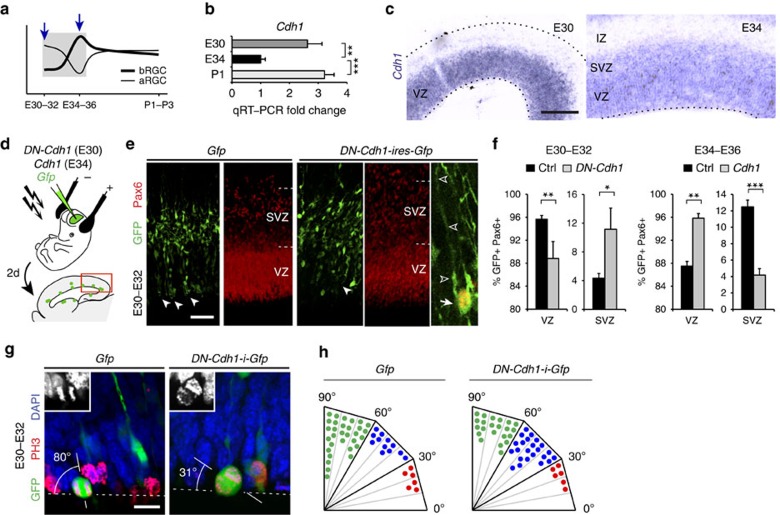
Onset of the restricted period for bRGC production and OSVZ generation depends on developmental variations of *Cdh1* expression. (**a**) Schematic of the early period of bRGC generation from aRGCs. (**b**,**c**) Candidate gene screening by qRT–PCR revealed *Cdh1* as differentially expressed in VZ between E30, E34 and P1 (**b**; mean values+s.e.m., **P<0.01, ***P<0.001, unpaired *t*-test), also shown by ISH (**c**). (**d**–**f**) *In utero* electroporation of *DN-Cdh1* from E30 to E32 increased bRGC production (GFP+/Pax6+ cells in SVZ) and decreased aRGCs, whereas overexpression of *Cdh1* from E34 to E36 had the opposite effect. In these experiments, GFP+ cells in SVZ were at high density, making unreliable the identification of bRGCs by morphology. Instead, bRGCs were identified as Pax6+/GFP+ cells with the cell body in SVZ, and aRGCs as Pax6+/GFP+ cells with the cell body in VZ and a distinct apical process. Numbers of cells and embryos analysed are indicated in Supplementary Table 4; *P<0.05, **P<0.01, ***P<0.001, *χ*^2^-test. (**g**,**h**) Analysis of cleavage orientation plane of VZ mitoses with respect to ventricular surface (dashed lines) in *Gfp*- and *DN-Cdh1-ires-Gfp*-overexpressing cells (*n*=103 cells, Ctrl; 51 cells, *DN-Cdh1*; 2 embryos per group). Insets are details of each mitosis under 4,6-diamidino-2-phenylindole (DAPI) stain. Each dot in **h** represents 2% of mitoses. Scale bar, 150 μm (**c**); 75 μm (**e**); 15 μm (**g**).

Unfortunately, functional gene annotation analysis of these gene sets showed that none of the ‘early' DEGs were associated to biological processes related to bRGC production, such as mitotic spindle orientation or cell delamination from the apical junction belt^7^,^19^,^37^ (Fig. 5b; Supplementary Fig. 5), and hence were not mechanistically promising. As microarray analysis always includes many false negatives due to the stringent statistical criteria, we scrutinized the array for AJ-related genes and found a trend of regulation for Cdh1. Therefore, we re-screened our samples by qRT–PCR to analyse AJ-related genes^30^. Among our tested candidates, *Cadherin1* (*Cdh1*) was the only gene with significantly lower messenger RNA levels in VZ at E34 compared with E30 (Fig. 5c). Low *Cdh1* levels are known to promote cell detachment from the VZ in the embryonic cerebral cortex^37^,^38^,^39^, so our data were consistent with the potential involvement of Cdh1 in regulating bRGC generation from aRGCs in VZ between E30 and E34 (Fig. 6a-c). To test this hypothesis, we first performed *in utero* electroporation to express a dominant-negative Cdh1 at E30 (DN-Cdh1, as described in ref. 40; Fig. 6d). Of note, due to the high density of GFP+ cells in SVZ on electroporation (Fig. 6e), bRGCs could not be identified by morphology, unlike with rv::*Gfp*; hence, aRGCs and bRGCs were identified here as GFP+ cells expressing Pax6+ in VZ and SVZ, respectively. Overexpression of *DN-Cdh1* caused a 2.5-fold increase in GFP+ bRGC abundance and a significant decrease in GFP+ aRGCs by E32 (Fig. 6e,f). Because only a proportion of aRGCs were electroporated, this decrease in GFP+ aRGCs did not cause a general depletion of the VZ. Conversely, electroporation to overexpress *Cdh1* at E34 was sufficient to abrogate the massive bRGC production normally occurring between E34 and E36, down to 33% of control embryos (Fig. 6f). Thus, reduced Cdh1 function is instrumental for the burst production of bRGCs destined for the OSVZ.

Cdh1 loss may promote bRGC formation and delamination by disruption of AJ integrity, but also by inducing changes in mitotic cleavage plane orientation, from vertical to oblique or horizontal^41^, as observed between E30 and E34 (Fig. 4e,f). To test this possibility, we compared the orientation of mitotic cleavage planes between control and cells expressing *DN-Cdh1* between E30 and E32. Similar to the endogenous reduction in cleavage angles between E30 and E34, we found that loss of Cdh1 function led to a switch from vertical to smaller angles (Fig. 6g,h). However, these changes did not perfectly phenocopy those observed between E32 and E34 in normal development (Fig. 4e), indicating that this process may be co-regulated by additional factors. These results demonstrated that the developmental loss of Cdh1 leading to increased bRGC production may act both on the switch in mitotic cleavage plane orientation and loss of cell adhesion.

**Figure 7:**
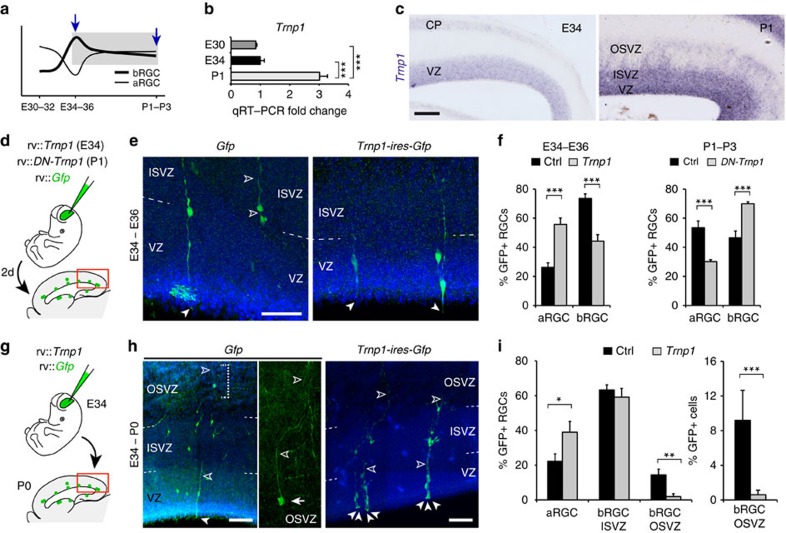
Closure of the restricted period for bRGC production and OSVZ generation depends on developmental variations of *Trnp1* expression. (**a**–**c**) Validation by qRT–PCR and ISH of differential expression of *Trnp1* between E34 and P1 (later period of bRGC generation from aRGC; mean values+s.e.m., ***P<0.001, unpaired *t*-test). (**d**–**f**) Overexpression of *Trnp1* by retroviral delivery from E34 to E36 decreased bRGC production and increased aRGC abundance, whereas expression of dominant-negative (DN) *Trnp1* from P1 to P3 had the opposite effect (mean values+s.e.m., ***P<0.001, *χ*^2^-test). (**g**–**i**) Sustained overexpression of *Trnp1* from E34 to P0 by retroviral delivery markedly blocked bRGC production for the OSVZ. Measures are relative to GFP+ RGCs, or all GFP+ cells in lineage (mean values+s.e.m., *P<0.05, **P<0.01, ***P<0.001, *χ*^2^-test). In all panels, solid arrowheads indicate apical end feet of aRGCs and open arrowheads indicate basal fibres of bRGCs. Scale bar, 150 μm (**c**); 75 μm (**e**,**h**).

Regarding ‘late' DEGs that might be responsible for the decrease in bRGC production between E34 and P1, Gene ontology analysis highlighted the terms cell adhesion, response to hormones, ions and steroids, organelle lumen, ribonucleotide binding and DNA repair (Fig. 5b). This list of ‘late' genes included *Trnp1*, which was expressed twofold more at P1 than at E34 (Supplementary Table 3; Supplementary Fig. 5; Supplementary Note 1). Trnp1 is a protein previously involved in bRGC generation, but only in mouse (without OSVZ), where acute downregulation of *Trnp1* causes overproduction of bRGCs^25^. Thus, our expression data suggested that low levels of endogenous *Trnp1* in ferret VZ at E34 may favour massive bRGC production for the OSVZ, while higher *Trnp1* levels at birth may close this period (Fig. 7a–c). To test the potential involvement of Trnp1 in regulating bRGC production for the OSVZ, we first used retroviruses to overexpress *Trnp1* in the VZ of E34 embryos and analysed the cellular output at E36 (Fig. 7d). The abundance of GFP+ bRGCs was reduced to nearly half in *Trnp1*-overexpressing embryos compared with control embryos expressing GFP alone, and this was concomitant with a twofold increase in aRGC abundance (Fig. 7e,f). This demonstrated that low levels of endogenous *Trnp1* expression are important for bRGC production between E34 and E36. To define if this decrease in bRGC generation indeed affects seeding of the OSVZ, we again overexpressed *Trnp1* in VZ cells at E34, but now followed by long-term survival until E42/P0 (Fig. 7g). The OSVZ of *Trnp1*-overexpressing ferrets was nearly devoid of GFP+ bRGCs (these only represented 0.9% of GFP+ cells), concomitant with a relative increase in aRGCs, but remarkably with no relative alteration of ISVZ (Fig. 7h,i). This demonstrated that in our previous short-survival experiments, many bRGCs observed in ISVZ were *en route* to the OSVZ. Also, that E34–E42/P0 is a period essential for the generation of bRGCs that will seed and found the OSVZ, with a peak of bRGC production between E34 and E36, before this layer becomes independent from VZ at P1.

Finally, we performed the converse manipulation, overexpressing a dominant-negative Trnp1 in VZ progenitors between P1 and P3 (Trnp1-GFP fusion protein, as described in ref. 25), to test whether the high endogenous levels of *Trnp1* expression at P1 might be responsible for the significant decrease in bRGC generation and closure of the restricted period (Fig. 7d). Compared with GFP-injected controls, expression of DN-Trnp1 increased significantly the production of bRGCs, while reducing aRGCs (Fig. 7f). Together, our results demonstrated that the dynamic temporal regulation of *Cdh1* and *Trnp1* expression is necessary and sufficient to control the variations in bRGC generation from aRGCs during cortical development. High expression of *Cdh1* and *Trnp1* is sufficient to limit bRGC generation before and after the restricted period, respectively, whereas low expression of both genes simultaneously is necessary for the massive self-consumption of aRGCs to produce bRGCs, and thus for the restricted period of OSVZ generation.

## Discussion

**Figure 8:**
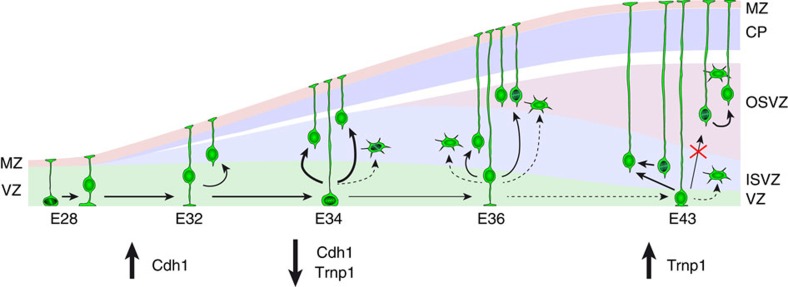
Model of developmental formation of OSVZ. During an early developmental period aRGCs self-renew, sustained by high levels of Cdh1. Between E34 and E36, a decrease in Cdh1 expression combined with low Trnp1 levels open the critical period for OSVZ formation, driving aRGCs into self-consuming divisions and generating bRGCs massively. These early bRGCs become founder cells of the OSVZ. After birth (E42/P0), a rise in Trnp1 levels blocks the production of bRGCs to the OSVZ and closes this critical period. From this point onward, the OSVZ follows a lineage independent from VZ and ISVZ, sustained by bRGC self-amplification, while VZ continues to seed bRGCs to ISVZ.

Our study identifies a completely novel mechanism involved in cortical development, whereby the formation of a germinal layer (OSVZ) depends on the seeding of founder progenitor cells during a restricted period. We show that the OSVZ first emerges by the accumulation of large amounts of bRGCs generated directly by apical progenitors in the VZ. This seeding of progenitor cells from VZ to OSVZ is only transient, and eventually the OSVZ lineage becomes completely independent from the other germinal layers and thereon relies on the self-amplification of its progenitor cells for further expansion to its remarkable size at subsequent stages (Fig. 8). These are highly unexpected findings, extending the complexity of the mechanisms that regulate cortical development and expansion^3^,^42^,^43^. We find that the period for bRGC seeding of the OSVZ depends on the combined temporal regulation of *Cdh1* and *Trnp1* expression in VZ (Fig. 8). These two genes were previously studied in mouse and shown to play key roles in the balance between self-renewal and delamination of aRGCs in the VZ^25^,^40^,^44^. Here we provide the first demonstration of a functional relationship between *Cdh1* and *Trnp1*, and of their endogenous regulation to dynamically modulate bRGC production in the developing embryo. Importantly, we show that abrogation of bRGC production by maintaining elevated Trnp1 levels from E34 onward resulted in a severe reduction of OSVZ cell lineages in the long term (E42/P0). Therefore, given that this period of production of bRGC for the OSVZ is very brief (with a maximum peak between E34 and E36 and very limited contributions at E36 and E38), its timing is under tight genetic control, and the abrogation of bRGC production during this embryonic period results in their virtual absence in the postnatal OSVZ, this may be considered a critical period for the formation of the OSVZ. Given the relevance of the OSVZ in the development of gyrencephaly^2^,^3^,^7^,^45^, the temporally dynamic regulation of endogenous *Trnp1* and *Cdh1* expression during embryogenesis might have evolved as a mechanism generating cortical phenotypic diversity in mammals.

Previous video microscopy analyses of slice cultures from embryonic human and non-human primate cerebral cortex revealed a wide variety of progenitor cell types in the OSVZ and other germinal layers, and some of their complex lineage relationships^8^,^10^,^29^. However, the developmental origin of the OSVZ during gestation was never identified primarily due to the lack of appropriate *in vivo* models where this can be addressed. Likewise, the types of neurons generated by VZ, ISVZ and OSVZ cannot be studied in slice cultures due to survival time limitations, but only *in vivo*. One previous report in the early postnatal ferret demonstrated that the lineages of both VZ and OSVZ are neurogenic, producing layer 2/3 pyramidal neurons with no reported differences^4^. Here we use also the ferret to define the cellular substrates for the developmental origin and expansion of the OSVZ *in vivo*. The process we have identified in ferret is completely different than in the mouse cerebral cortex, where basal progenitors rarely self-renew and the SVZ is formed by transient populations of progenitor cells that are continuously seeded by the VZ and self-consumed shortly after^13^,^14^,^15^,^16^,^24^. Even in the mouse lateral ganglionic eminence, where the SVZ is largest and progenitors frequently self-amplify, there is continuous seeding from aRGCs^26^. Importantly, although rodents and primates are closer in phylogeny than with carnivores such as the ferret^46^, many of the typical features of OSVZ and bRGCs in ferret are strikingly similar to human and macaque, but different from mouse (that is, self-amplification, relative abundance of bRGCs and IPCs). Hence, we speculate that a similar critical period for the formation of the OSVZ, and its subsequent independent lineage, may also occur in human and other primates. Given that seeding of the OSVZ occurs only during a brief and transient period (E34–P0), the rate of self-amplification of its constituent progenitor cells becomes a major factor for its expansion and size. This notion is consistent with the dynamics of bRGC proliferation in the OSVZ observed in non-human primates, where this layer undergoes a massive increase in the size during a period of rapid bRGC self-amplification^8^. Intriguingly, the ISVZ seems to share features with the mouse SVZ, as it continuously receives bRGCs from the VZ while maintaining some self-renewing capacity. Expansive cell lineages are frequent in the mouse lateral ganglionic eminence (LGE)^26^, suggesting the evolutionary co-option of this strategy in forebrain development^42^.

One major difference between aRGCs and bRGCs is their epithelial nature, where aRGCs are anchored to the ventricular surface by AJs, while bRGCs are not. Thus, the generation of bRGCs from aRGCs may be favoured by oblique and horizontal mitotic cleavage planes, combined with an active loss of AJs, leading to the apical detachment of daughter cells^17^,^25^,^26^,^27^,^29^. Orientation of mitotic spindle is molecularly regulated by a protein complex including mInsc, LGN, Par3, aPKC and also Cdh1, the blockade of which favours oblique or horizontal cleavage planes and results in an excess of daughter cells delaminating from the ventricular surface^33^,^41^,^47^,^48^. Accordingly, we find that the onset of the restricted period with a massive generation of bRGCs from aRGCs is bookmarked by a significant decrease in *Cdh1* expression and a shift in mitotic cleavage plane orientation, and we have demonstrated a causal relationship between these three events. However, the combined frequency of divisions with oblique or horizontal cleavage planes that we have observed at the critical period (E34: 50%) can only explain the delamination from VZ of 25% of daughter cells, but not 70% as we observed by lineage tracing (Fig. 4). The delamination of additional cells may require the loss of apical anchoring, which occurs on the loss of AJ proteins, their links to the cytoplasm such as α- and β-catenin or regulators of the F-actin belt linking AJs such as RhoA^30^,^49^. Among these factors, Cdh1 is a crucial component of AJs previously implicated in regulating apical cell delamination (though not bRGC production), and here we show that the loss of Cdh1 leads to the loss of aRGCs and increased bRGC production. Hence, our present findings point at Cdh1 as key in the production of bRGCs from aRGCs by regulating both mitotic cleavage plane orientation and delamination from the AJ belt. Repression of cadherins and cell delamination from VZ is promoted by the Snail family of transcription factors, and also by Robo receptor signalling^37^,^38^,^50^. Unfortunately, current detection methods are insufficient to reveal differences in membrane-bound Cdh1 between individual aRGCs primed for delamination versus not. Finally, closure of the period of massive bRGC production at E36 is marked by an increase in *Trnp1* expression, which favours vertical cleavage planes and has been shown to repress bRGC production in mouse^25^.

Our results demonstrate that the combined regulation of endogenous *Cdh1* and *Trnp1* levels is necessary and sufficient to control the abundance of VZ cell depletion and bRGC seeding, and thus it defines the time window for our newly identified critical period of OSVZ formation. Our Cdh1 and Trnp1 downregulation experiments demonstrate that aRGCs have the intrinsic potential to generate OSVZ bRGCs before and after the critical period, so the onset and duration of this period could be easily modified in phylogeny or disease by changing the temporal expression of these genes. Given the relevance of the OSVZ in cortical expansion and folding^2^,^3^,^7^, the evolution of mechanisms controlling the precise temporal regulation of critical period genes seems key to generate the extraordinary diversity of cortical phenotypes across mammals^42^,^51^.

Critical periods are unique windows of opportunity during development, but also periods of high vulnerability to disease^52^,^53^. Given the central role of the OSVZ in cortical development from ferrets to monkeys and humans, including neurogenesis and surface area expansion^4^,^6^,^8^,^10^, the critical period of OSVZ seeding is a time of susceptibility to cortical disease, where subtle or acute defects may have magnified long-term consequences. Indeed, acute abrogation of cell proliferation in ferret embryos at the onset of this critical period leads to reduced OSVZ and lissencephaly^54^. In humans, brain malformations due to acute insults in fetal development are rarely traced, but defects in VZ integrity, progenitor proliferation and precise gene expression regulation are emerging as critical in malformations of cortical development^55^,^56^,^57^,^58^. Our identification of the critical period for OSVZ formation in ferret brings a novel perspective to fundamental mechanisms of cortical development, and this may help to better frame the mechanistic effect of mutations perturbing cerebral cortex development in humans.

## Methods

### Animals

Pigmented ferrets (*Mustela putorius furo*) were obtained from Marshall Bioresources (North Rose, NY) and isoquimen (Barcelona, Spain), and kept on a 16:8-h light:dark cycle at the Animal Facilities of the Universidad Miguel Hernández. Wild-type mice were maintained in an Institute for Cancer Research; Harlan Inc. (ICR) background. The day of vaginal plug was considered as embryonic day (E) 0.5. All animals were treated according to Spanish and EU regulations, and experimental protocols were approved by the Universidad Miguel Hernández Institutional Animal Care and Use Committee (IACUC).

### Constructs

For retroviral delivery, constructs encoding *Gfp* alone, *Trnp1*-*Gfp* fusion protein (Trnp1-DN) or a bicistronic cassette encoding *Trnp1*-IRES-*Gfp*^25^ were subcloned into an murine moloney leukemia virus (MMLV) retroviral-packaging vector downstream of the CAG promoter (generous gift of F.H. Gage). For electroporation, constructs encoding *Gfp*, *E-Cadherin* (Addgene #28009) or *DN-Cdh1* ref. 40) were subcloned into a pCAG promoter-containing vector. All plasmids were produced under endotoxin-free conditions (QIAGEN EndoFree Plasmid Maxi kit).

### Virus injections and electroporation

High-titre MMLV-based VSVG-pseudotyped retrovirus (5 × 10^7^–5 × 10^8^ p.f.u. ml^−1^) encoding *Gfp*, *Trnp1-ires-Gfp* or *Trnp1-Gfp* under the CAG promoter were prepared by transient transfection of HEK293 cells, concentrated by ultracentrifugation and viral titre estimated by clonal infection of HEK cell cultures^59^. High-titre replication-incompetent adenovirus (9.3 × 10^11^ v.p. ml^−1^) encoding *Gfp* under the CMV promoter were obtained from QBioGene (Irvine, CA). Viral solutions were injected using pulled glass micropippettes. For postnatal injections, ferret kits were deeply anaesthetized and maintained with 1.5% isoflurane during surgery, and injections were aimed at the telencephalic lateral ventricle, ISVZ or OSVZ by means of stereotaxic coordinates: lateral ventricle: antero-posterior (AP)=−0.5 mm, latero-medial (LM)=2.0 mm, dorso-ventral (DV)=2.0 mm, with an AP inclination of 22.5°; ISVZ: AP=−1.3 mm, LM=2.0 mm, DV=2.0 mm, with an AP inclination of 22.5°; OSVZ: AP=−1.3 mm, LM=1.6 mm, DV=2 mm, with an AP inclination of 45°. These injections were very accurate, as confirmed *a posteriori* with short-survival animals where the bulk of GFP-labelled cells indicated the tip of the injection pipette (for ISVZ and OSVZ injections), and sparse cell labelling in VZ across the entire cortex indicated that the virus had been injected in the lateral telencephalic ventricle (Fig. 1). We only found 2 out of 11 animals where injections aimed at ISVZ actually labelled a sparse number of VZ cells, and thus had occurred in the ventricular cavity. On injection in ISVZ and OSVZ, aRGCs cells in VZ and bRGCs in ISVZ were also labelled immediately below the injection site, infected retrogradely via their basal process.

For *in utero* injections in both ferret and mouse, timed-pregnant females were deeply anaesthetized and maintained in 2% isoflurane during surgery. The abdominal cavity was open, the uterine horns exposed and retrovirus solutions were pressure injected into the telencephalic lateral ventricle of embryos through the uterine wall.

For electroporation, DNA plasmids encoded *Gfp*, *E-Cadherin* (*Cdh1*) or *DN-Cdh1*, under the CAG promoter. *In utero* and postnatal electroporation of ferret embryos and kits were performed as described^60^,^61^. After the appropriate survival period, postnatal kits or pregnant females were overdosed with sodium pentobarbital (Nembutal), and further processed for immunohistochemistry or *in situ* hybridization (ISH).

### Immunohistochemistry and ISH

For histological analysis, embryos were obtained by caesarean section of timed-pregnant females on deep anaesthesia with sodium pentobarbital, and perfused transcardially with 4% paraformaldehyde; postnatal ferrets were deeply anaesthetized with sodium pentobarbital before transcardiac perfusion with paraformaldehyde. After perfusion, brains were extracted and sectioned. For immunohistochemistry, brain sections were incubated with primary antibodies overnight (anti-GFP, 1:1,000, Aves Labs; anti-Pax6 1:500, Millipore; anti-Tbr2 1:200, Abcam; anti-PhVim, 1:1,000, Abcam; anti-PH3, 1:1,000, Upstate; anti-Ki67, 1:200, Novocastra; anti-BrdU, 1:200, Abcam; anti-GFAP, 1:1,000, Dako; anti-Olig2, 1:100, IBL), followed by appropriate fluorescently conjugated secondary antibodies (1:200, Jackson), and counterstained with 4,6-diamidino-2-phenylindole (Sigma). For anti-BrdU staining, sections were pretreated with 2 N HCl for 30 min. For ISH, sense and anti-sense complementary RNA probes were synthesized and labelled with digoxigenin (Roche Diagnostics) according to the manufacturer's instructions. ISH was performed as described previously^4^. Ferret-specific ISH probes were cloned using the following primers: *Trnp1*, forward: 5′- TCTGGACTCCTGGATTGAGC -3′, reverse: 5′- TGCCGCTGTGTCTATCTGAG -3′; *Cdh1*, forward: 5′- CGGAGCTGAGTTTTCTGGTC -3′, reverse: 5′- GAGGCTGTGGATTCTTCTGG -3′. These ferret-specific primers were designed based on the same ferret-specific sequences as in the microarray. PCR was performed using Go Taq Flexi DNA polymerase (Promega), and the resulting amplicons were purified with Wizard SV Gel and PCR Clean-Up System (Promega) and cloned into pGEM-T Easy Vector System I.

### Slice culture and time-lapse imaging

Ferret brain slices were prepared and maintained in culture as described previously^62^. To image aRGC behaviours, P1 ferrets were injected with rv::*Gfp*, and their brains obtained for slicing at P6. To image the behaviour of bRGCs in OSVZ, slices were prepared from unmanipulated P18 ferrets, and 1 h later, slices received various injections of Ad-GFP (3–6 nl per injection site, 2–8 injection sites per slice) in the cortical plate. One day after slice preparation, slices containing fluorescent cells were selected for time-lapse imaging. Images were obtained either under fluorescein optics (Filter 41017, EX449-489/EM500-548; Chroma, Rockingham, VT, USA) through an air immersion × 20 lens in an inverted epifluorescence microscope or under two-photon optics (× 40) through a water immersion × 20 lens in a Leica SP2 inverted microscope, both equipped with an incubation chamber: 5% CO_2_, 37 °C. Frames were obtained intermittently during 49–142 h in culture. Digital images were acquired, contrast-enhanced and analysed with Metamorph (Microbrightfield) or Imaris software (Bitplane).

### Microarray and qRT–PCR

For RNA extraction, P1 ferret kits were deeply anaesthetized, and timed-pregnant ferret females were deeply anaesthetized and their living embryos were obtained by caesarean section. Subjects were then decapitated, their brains dissected and blocked in ice-cold ACSF (140 mM NaCl, 5 mM KCl, 1 mM MgCl_2_, 24 mM D-glucose, 10 mM HEPES, 1 mM CaCl_2_, pH 7.2), and tissue blocks containing the occipital cortex were vibrotome-cut in 300-μm-thick slices. Living cortical slices were further microdissected with microscalpels in ice-cold ACSF to isolate the VZ from the caudal pole of the cerebral cortex. Germinal layers were identified in living slices under the dissection scope, where the VZ was the most opaque layer on the apical side of the cortex. Tissue pieces were fresh-frozen in Trizol for RNA extraction, with a post-mortem interval of <1 h.

Total RNA was extracted using RNeasy Mini kit (Quiagen), followed by treatment with RNase-Free DNase Set (Quiagen). RNA quality was confirmed using the RNA 6000 Nano kit on the Agilent Bioanalyzer platform, and then 200 ng of total RNA was labelled using the one-colour labelling kit from Agilent Technologies according to the manufacturer's protocol. Labelled complementary RNA was then hybridized for 16 h on a custom-made microarray containing 43,692 ferret-specific probes covering 17,386 genes^63^. Microarray slides were scanned on an Agilent High-Resolution C Scanner, and the raw image files were processed by the Agilent feature extraction software. Raw data files were normalized using quantile normalization in Partek Genomics Suite. Statistical analysis of microarray data was done in Multiexperiment Viewer^64^. To identify genes with significantly different expression levels, we used analysis of variance comparisons between samples, using *P*-values based on 500 permutations and Bonferroni false discovery correction, as in Ayoub *et al*.^65^. Functional gene annotation analysis was performed using the web-based DAVID v6.7 software (http://david.abcc.ncifcrf.gov)^66^. The microarray data from this publication have been submitted to the GEO database (http://www.ncbi.nlm.nih.gov/geo/) and assigned the identifier GSE63203.

For qRT–PCR, primers for ferret gene homologues were designed based on the same ferret-specific sequences. Template complementary DNA was generated using Maxima First Strand cDNA Synthesis kit for quantitative real-time PCR (qRT–PCR; Thermo Fisher). Quantitative RT–PCR was performed using the Step One Plus sequence detection system and the SYBR Green method (Applied Biosystems), with each point examined in triplicate. Transcript levels were calculated using the comparative Ct method normalized using actin. Primers used were: Trnp1, forward: 5′- TTGGTCTGAGAAATCCCTGC -3′, reverse: 5′- CGCTGTGTCTATCTGAGGAAG -3′; Cdh1, forward: 5′- TGCCCAGAAAACGAGAAAGG -3′, reverse: 5′- ACAAATACACCAACCGGAGG -3′; actin, primers as in ref. 67. In each experimental group, we analysed two to three samples, each consisting of a pool of two to three embryos/kits. Reactions were performed in triplicate per independent sample. Data were statistically analysed with SPSS software using *t*-test.

### Cell count measurements

Cell types were identified according to the following criteria: aRGC, cell with the soma located within the VZ, a single apical process contacting the ventricular surface and a very long radially oriented basal process, expressing Pax6 and Ki67; bRGC, cell with the soma located outside the VZ, with a very long radially oriented basal process, without an apical process anchored to the ventricular AJ belt, expressing Pax6 and Ki67; MP, cell with multiple short processes extended from the cell soma and without obvious polarity; IPC, MP cell expressing Ki67 and Tbr2; migrating neuron, cell with clear apical–basal polarity extending a relatively thin basal process much shorter than that of RGCs, and a much shorter and thinner apical process; DN, cell with a single basally-directed process highly branched at a distance from the cell body, frequently located at the top of the cortical plate; StC, multipolar cell with several very highly branched and short processes extending from the cell soma. In electroporation experiments, identification of GFP+ cell identity in SVZ based on morphology was unreliable due to their high density. In those experiments, aRGCs were defined as Pax6+/GFP+ cells with the cell body in VZ and a distinct apical process, while bRGCs were identified as Pax6+/GFP+ cells with the cell body in SVZ. The angle of mitotic cleavage plane was measured using PH3 stains and considering only cells at telophase. Angles were measured with respect to the general trajectory of the radial fibre scaffold, or with respect to the ventricular surface^20^,^27^. Cleavage planes were considered horizontal if they occurred at 60–90° with respect to radial fibres or 0–30° with respect to the ventricular surface.

### Double-labelling analyses

Quantification of cell co-staining was performed by confocal microscopy (Leica) through a × 40 lens and × 2–4 zoom. Images were acquired from cells in four sections per subject, two to three subjects per condition and age. Images were analysed using Imaris software (Bitplane) and Canvas X software.

### Progenitor self-amplification

To determine whether the population of RGCs increased or not, we profited from a unique property of retroviruses: the GFP reporter gene they encode is randomly integrated into one of the two daughter cells after division of the originally infected cell. Thus, if a bRGC infected with our retrovirus generates two bRGCs, one will always observe one GFP+ bRGC; but if it divides to generate one bRGC+one neuron, there is a random chance of observing the bRGC or the neuron (50–50%). This unique property of retroviruses has been utilized previously by other labs^13^,^68^,^69^. Taken to the population level, in a homogeneous pool of symmetric amplificative divisions where each progenitor cell generates two progenitors like itself, 100% of GFP+ cells will be progenitors; in a homogeneous population of asymmetric divisions, where each progenitor divides to generate one progenitor like itself plus a different cell, 50% of labelled cells will be progenitors and 50% will be of another type; in a homogeneous population of symmetric self-consuming divisions, where progenitors divide to generate two cells different than the mother, 0% of labelled cells will be like the mother progenitor. Therefore, if after one cell division we observed that >50% of GFP+ cells were bRGCs, like in our P1–P3 and P6–P8 experiments, this means that the population of bRGCs was expanding (there was a net production). Unfortunately, it is not possible to know with precision which kinds of divisions occurred, but one can conclude an increase in this cell type at the population level.

### Statistical analysis

Statistical analysis was performed using either unpaired *t*-test (two-tailed distribution) or χ^2^-test. For analysis of microarray data, we performed one-way analysis of variance followed by Bonferroni's test for multiple comparisons and Duncan's test for subset homogeneity. All values represent mean values±s.e.m. Normality and equality of variance were formally tested with SPSS Statistics software.

### Data availability

The microarray data that support the findings of this study have been deposited in the GEO database (http://www.ncbi.nlm.nih.gov/geo/) and assigned the identifier GSE63203. All other relevant data are available from the authors.

## Additional information

**How to cite this article:** Martínez-Martínez, M. A. *et al*. A restricted period for formation of outer subventricular zone defined by Cdh1 and Trnp1 levels. *Nat. Commun.* 7:11812 doi: 10.1038/ncomms11812 (2016).

## Supplementary Material

Supplementary Information Supplementary Figures 1 - 5, Supplementary Tables 1 - 4, Supplementary Note 1 and Supplementary References

## References

[b1] Smart I. H., Dehay C., Giroud P., Berland M. & Kennedy H. Unique morphological features of the proliferative zones and postmitotic compartments of the neural epithelium giving rise to striate and extrastriate cortex in the monkey. Cereb. Cortex 12, 37–53 (2002). 11734531 10.1093/cercor/12.1.37 PMC1931430

[b2] Borrell V. & Reillo I. Emerging roles of neural stem cells in cerebral cortex development and evolution. Dev. Neurobiol. 72, 955–971 (2012). 22684946 10.1002/dneu.22013

[b3] Lui J. H., Hansen D. V. & Kriegstein A. R. Development and evolution of the human neocortex. Cell 146, 18–36 (2011). 21729779 10.1016/j.cell.2011.06.030 PMC3610574

[b4] Reillo I., de Juan Romero C., Garcia-Cabezas M. A. & Borrell V. A role for intermediate radial glia in the tangential expansion of the mammalian cerebral cortex. Cereb. Cortex 21, 1674–1694 (2011). 21127018 10.1093/cercor/bhq238

[b5] Bayatti N. . A molecular neuroanatomical study of the developing human neocortex from 8 to 17 postconceptional weeks revealing the early differentiation of the subplate and subventricular zone. Cereb. Cortex 18, 1536–1548 (2008). 17965125 10.1093/cercor/bhm184 PMC2430151

[b6] Nonaka-Kinoshita M. . Regulation of cerebral cortex size and folding by expansion of basal progenitors. EMBO J. 32, 1817–1828 (2013). 23624932 10.1038/emboj.2013.96 PMC3926188

[b7] Borrell V. & Gotz M. Role of radial glial cells in cerebral cortex folding. Curr. Opin. Neurobiol. 27, 39–46 (2014). 24632307 10.1016/j.conb.2014.02.007

[b8] Betizeau M. . Precursor diversity and complexity of lineage relationships in the outer subventricular zone of the primate. Neuron 80, 442–457 (2013). 24139044 10.1016/j.neuron.2013.09.032

[b9] Fietz S. A. . OSVZ progenitors of human and ferret neocortex are epithelial-like and expand by integrin signaling. Nat. Neurosci. 13, 690–699 (2010). 20436478 10.1038/nn.2553

[b10] Hansen D. V., Lui J. H., Parker P. R. & Kriegstein A. R. Neurogenic radial glia in the outer subventricular zone of human neocortex. Nature 464, 554–561 (2010). 20154730 10.1038/nature08845

[b11] Kowalczyk T. . Intermediate neuronal progenitors (basal progenitors) produce pyramidal-projection neurons for all layers of cerebral cortex. Cereb. Cortex 19, 2439–2450 (2009). 19168665 10.1093/cercor/bhn260 PMC2742596

[b12] Englund C. . Pax6, Tbr2, and Tbr1 are expressed sequentially by radial glia, intermediate progenitor cells, and postmitotic neurons in developing neocortex. J. Neurosci. 25, 247–251 (2005). 15634788 10.1523/JNEUROSCI.2899-04.2005 PMC6725189

[b13] Noctor S. C., Martinez-Cerdeno V., Ivic L. & Kriegstein A. R. Cortical neurons arise in symmetric and asymmetric division zones and migrate through specific phases. Nat. Neurosci. 7, 136–144 (2004). 14703572 10.1038/nn1172

[b14] Haubensak W., Attardo A., Denk W. & Huttner W. B. Neurons arise in the basal neuroepithelium of the early mammalian telencephalon: a major site of neurogenesis. Proc. Natl Acad. Sci. USA 101, 3196–3201 (2004). 14963232 10.1073/pnas.0308600100 PMC365766

[b15] Miyata T. . Asymmetric production of surface-dividing and non-surface-dividing cortical progenitor cells. Development 131, 3133–3145 (2004). 15175243 10.1242/dev.01173

[b16] Attardo A., Calegari F., Haubensak W., Wilsch-Brauninger M. & Huttner W. B. Live imaging at the onset of cortical neurogenesis reveals differential appearance of the neuronal phenotype in apical versus basal progenitor progeny. PLoS ONE 3, e2388 (2008). 18545663 10.1371/journal.pone.0002388 PMC2398773

[b17] Shitamukai A., Konno D. & Matsuzaki F. Oblique radial glial divisions in the developing mouse neocortex induce self-renewing progenitors outside the germinal zone that resemble primate outer subventricular zone progenitors. J. Neurosci. 31, 3683–3695 (2011). 21389223 10.1523/JNEUROSCI.4773-10.2011 PMC6622781

[b18] Wang X., Tsai J. W., Lamonica B. & Kriegstein A. R. A new subtype of progenitor cell in the mouse embryonic neocortex. Nat. Neurosci. 14, 555–561 (2011). 21478886 10.1038/nn.2807 PMC3083489

[b19] Taverna E., Gotz M. & Huttner W. B. The cell biology of neurogenesis: toward an understanding of the development and evolution of the neocortex. Annu. Rev. Cell Dev. Biol. 30, 465–502 (2014). 25000993 10.1146/annurev-cellbio-101011-155801

[b20] Reillo I. & Borrell V. Germinal zones in the developing cerebral cortex of ferret: ontogeny, cell cycle kinetics, and diversity of progenitors. Cereb. Cortex 22, 2039–2054 (2012). 21988826 10.1093/cercor/bhr284

[b21] Garcia-Moreno F., Vasistha N. A., Trevia N., Bourne J. A. & Molnar Z. Compartmentalization of cerebral cortical germinal zones in a lissencephalic primate and gyrencephalic rodent. Cereb. Cortex 22, 482–492 (2012). 22114081 10.1093/cercor/bhr312

[b22] Kelava I. . Abundant occurrence of basal radial glia in the subventricular zone of embryonic neocortex of a lissencephalic primate, the common marmoset *Callithrix jacchus*. Cereb Cortex 22, 469–481 (2012). 22114084 10.1093/cercor/bhr301 PMC3256412

[b23] Martinez-Cerdeno V. . Comparative analysis of the subventricular zone in rat, ferret and macaque: evidence for an outer subventricular zone in rodents. PLoS One 7, e30178 (2012). 22272298 10.1371/journal.pone.0030178 PMC3260244

[b24] Noctor S. C., Martinez-Cerdeno V. & Kriegstein A. R. Distinct behaviors of neural stem and progenitor cells underlie cortical neurogenesis. J. Comp. Neurol. 508, 28–44 (2008). 18288691 10.1002/cne.21669 PMC2635107

[b25] Stahl R. . Trnp1 regulates expansion and folding of the mammalian cerebral cortex by control of radial glial fate. Cell 153, 535–549 (2013). 23622239 10.1016/j.cell.2013.03.027

[b26] Pilz G. A. . Amplification of progenitors in the mammalian telencephalon includes a new radial glial cell type. Nat. Commun. 4, 2125 (2013). 23839311 10.1038/ncomms3125 PMC3717501

[b27] Gertz C. C., Lui J. H., LaMonica B. E., Wang X. & Kriegstein A. R. Diverse behaviors of outer radial glia in developing ferret and human cortex. J. Neurosci. 34, 2559–2570 (2014). 24523546 10.1523/JNEUROSCI.2645-13.2014 PMC3921426

[b28] Jackson C. A., Peduzzi J. D. & Hickey T. L. Visual cortex development in the ferret. I. Genesis and migration of visual cortical neurons. J. Neurosci. 9, 1242–1253 (1989). 2703875 10.1523/JNEUROSCI.09-04-01242.1989 PMC6569864

[b29] LaMonica B. E., Lui J. H., Hansen D. V. & Kriegstein A. R. Mitotic spindle orientation predicts outer radial glial cell generation in human neocortex. Nat. Commun. 4, 1665 (2013). 23575669 10.1038/ncomms2647 PMC3625970

[b30] Gotz M. & Huttner W. B. The cell biology of neurogenesis. Nat. Rev. Mol. Cell Biol. 6, 777–788 (2005). 16314867 10.1038/nrm1739

[b31] Turrero Garcia M., Chang Y., Arai Y. & Huttner W. B. S-phase duration is the main target of cell cycle regulation in neural progenitors of developing ferret neocortex. J. Comp. Neurol. 524, 456–470 (2015). 25963823 10.1002/cne.23801 PMC5008145

[b32] Xie Y., Juschke C., Esk C., Hirotsune S. & Knoblich J. A. The phosphatase PP4c controls spindle orientation to maintain proliferative symmetric divisions in the developing neocortex. Neuron 79, 254–265 (2013). 23830831 10.1016/j.neuron.2013.05.027 PMC3725415

[b33] Postiglione M. P. . Mouse inscuteable induces apical-basal spindle orientation to facilitate intermediate progenitor generation in the developing neocortex. Neuron 72, 269–284 (2011). 22017987 10.1016/j.neuron.2011.09.022 PMC3199734

[b34] Bruder C. E. . Transcriptome sequencing and development of an expression microarray platform for the domestic ferret. BMC Genomics 11, 251 (2010). 20403183 10.1186/1471-2164-11-251 PMC2873475

[b35] de Juan Romero C., Bruder C., Tomasello U., Sanz-Anquela J. M. & Borrell V. Discrete domains of gene expression in germinal layers distinguish the development of gyrencephaly. EMBO J. 34, 1859–1874 (2015). 25916825 10.15252/embj.201591176 PMC4547892

[b36] Janz R., Goda Y., Geppert M., Missler M. & Sudhof T. C. SV2A and SV2B function as redundant Ca2+ regulators in neurotransmitter release. Neuron 24, 1003–1016 (1999). 10624962 10.1016/s0896-6273(00)81046-6

[b37] Itoh Y. . Scratch regulates neuronal migration onset via an epithelial-mesenchymal transition-like mechanism. Nat. Neurosci. 16, 416–425 (2013). 23434913 10.1038/nn.3336

[b38] Borrell V. . Slit/Robo signaling modulates the proliferation of central nervous system progenitors. Neuron 76, 338–352 (2012). 23083737 10.1016/j.neuron.2012.08.003 PMC4443924

[b39] Hatakeyama J. . Cadherin-based adhesions in the apical endfoot are required for active Notch signaling to control neurogenesis in vertebrates. Development 141, 1671–1682 (2014). 24715457 10.1242/dev.102988

[b40] Noles S. R. & Chenn A. Cadherin inhibition of beta-catenin signaling regulates the proliferation and differentiation of neural precursor cells. Mol. Cell. Neurosci. 35, 549–558 (2007). 17553695 10.1016/j.mcn.2007.04.012

[b41] Le Borgne R., Bellaiche Y. & Schweisguth F. Drosophila E-cadherin regulates the orientation of asymmetric cell division in the sensory organ lineage. Curr. Biol. 12, 95–104 (2002). 11818059 10.1016/s0960-9822(01)00648-0

[b42] Borrell V. & Calegari F. Mechanisms of brain evolution: Regulation of neural progenitor cell diversity and cell cycle length. Neurosci. Res. 86, 14–24 (2014). 24786671 10.1016/j.neures.2014.04.004

[b43] Rakic P. Evolution of the neocortex: a perspective from developmental biology. Nat. Rev. Neurosci. 10, 724–735 (2009). 19763105 10.1038/nrn2719 PMC2913577

[b44] Rasin M. R. . Numb and Numbl are required for maintenance of cadherin-based adhesion and polarity of neural progenitors. Nat. Neurosci. 10, 819–827 (2007). 17589506 10.1038/nn1924

[b45] Sun T. & Hevner R. F. Growth and folding of the mammalian cerebral cortex: from molecules to malformations. Nat. Rev. Neurosci. 15, 217–232 (2014). 24646670 10.1038/nrn3707 PMC4107216

[b46] Bininda-Emonds O. R. . The delayed rise of present-day mammals. Nature 446, 507–512 (2007). 17392779 10.1038/nature05634

[b47] Morin X., Jaouen F. & Durbec P. Control of planar divisions by the G-protein regulator LGN maintains progenitors in the chick neuroepithelium. Nat. Neurosci. 10, 1440–1448 (2007). 17934458 10.1038/nn1984

[b48] Inaba M., Yuan H., Salzmann V., Fuller M. T. & Yamashita Y. M. E-cadherin is required for centrosome and spindle orientation in *Drosophila* male germline stem cells. PLoS ONE 5, e12473 (2010). 20824213 10.1371/journal.pone.0012473 PMC2930853

[b49] Cappello S. . A radial glia-specific role of RhoA in double cortex formation. Neuron 73, 911–924 (2012). 22405202 10.1016/j.neuron.2011.12.030

[b50] Wong G. K., Baudet M. L., Norden C., Leung L. & Harris W. A. Slit1b-Robo3 signaling and N-cadherin regulate apical process retraction in developing retinal ganglion cells. J. Neurosci. 32, 223–228 (2012). 22219284 10.1523/JNEUROSCI.2596-11.2012 PMC3272413

[b51] Welker W. in *Cerebral Cort*ex (eds Peters A. & Jones E.G.) (Plenum Press, 1990).

[b52] LeBlanc J. J. & Fagiolini M. Autism: a "critical period" disorder? Neural. Plast. 2011, 921680 (2011). 21826280 10.1155/2011/921680 PMC3150222

[b53] Levelt C. N. & Hubener M. Critical-period plasticity in the visual cortex. Annu. Rev. Neurosci. 35, 309–330 (2012). 22462544 10.1146/annurev-neuro-061010-113813

[b54] Poluch S. & Juliano S. L. Fine-tuning of neurogenesis is essential for the evolutionary expansion of the cerebral cortex. Cereb. Cortex 25, 346–364 (2013). 23968831 10.1093/cercor/bht232 PMC4351427

[b55] Kielar M. . Mutations in Eml1 lead to ectopic progenitors and neuronal heterotopia in mouse and human. Nat. Neurosci. 17, 923–933 (2014). 24859200 10.1038/nn.3729

[b56] Bae B. I. . Evolutionarily dynamic alternative splicing of GPR56 regulates regional cerebral cortical patterning. Science 343, 764–768 (2014). 24531968 10.1126/science.1244392 PMC4480613

[b57] Cappello S. . Mutations in genes encoding the cadherin receptor-ligand pair DCHS1 and FAT4 disrupt cerebral cortical development. Nat. Genet. 45, 1300–1308 (2013). 24056717 10.1038/ng.2765

[b58] Barkovich A. J., Guerrini R., Kuzniecky R. I., Jackson G. D. & Dobyns W. B. A developmental and genetic classification for malformations of cortical development: update 2012. Brain 135, 1348–1369 (2012). 22427329 10.1093/brain/aws019 PMC3338922

[b59] Tashiro A., Zhao C. & Gage F. H. Retrovirus-mediated single-cell gene knockout technique in adult newborn neurons in vivo. Nat. Protoc. 1, 3049–3055 (2006). 17406567 10.1038/nprot.2006.473

[b60] Borrell V. In vivo gene delivery to the postnatal ferret cerebral cortex by DNA electroporation. J. Neurosci. Methods 186, 186–195 (2010). 19944720 10.1016/j.jneumeth.2009.11.016

[b61] Kawasaki H., Toda T. & Tanno K. *In vivo* genetic manipulation of cortical progenitors in gyrencephalic carnivores using in utero electroporation. Biol. Open 2, 95–100 (2013). 23336081 10.1242/bio.20123160 PMC3545273

[b62] Borrell V., Kaspar B. K., Gage F. H. & Callaway E. M. *In vivo* evidence for radial migration of neurons by long-distance somal translocation in the developing ferret visual cortex. Cereb. Cortex 16, 1571–1583 (2006). 16357334 10.1093/cercor/bhj094

[b63] Camp J. V. . *De-novo* transcriptome sequencing of a normalized cDNA pool from influenza infected ferrets. PLoS One 7, e37104 (2012). 22606336 10.1371/journal.pone.0037104 PMC3350496

[b64] Saeed A. I. . TM4: a free, open-source system for microarray data management and analysis. Biotechniques 34, 374–378 (2003). 12613259 10.2144/03342mt01

[b65] Ayoub A. E. . Transcriptional programs in transient embryonic zones of the cerebral cortex defined by high-resolution mRNA sequencing. Proc. Natl Acad. Sci. USA 108, 14950–14955 (2011). 21873192 10.1073/pnas.1112213108 PMC3169109

[b66] Dennis G. Jr. . DAVID: Database for Annotation, Visualization, and Integrated Discovery. Genome Biol. 4, P3 (2003). 12734009 PMC193660

[b67] Fang Y. . Molecular characterization of in vivo adjuvant activity in ferrets vaccinated against influenza virus. J. Virol. 84, 8369–8388 (2010). 20534862 10.1128/JVI.02305-09 PMC2919000

[b68] Brown K. N. . Clonal production and organization of inhibitory interneurons in the neocortex. Science 334, 480–486 (2011). 22034427 10.1126/science.1208884 PMC3304494

[b69] Ciceri G. . Lineage-specific laminar organization of cortical GABAergic interneurons. Nat. Neurosci. 16, 1199–1210 (2013). 23933753 10.1038/nn.3485

