# Supplementary material for: A restricted period for formation of outer subventricular zone defined by Cdh1 and Trnp1 levels
Source: Nat Commun. 2016 Jun 6;7:11812. doi: 10.1038/ncomms11812 (PMC4897765; doi:10.1038/ncomms11812)
Supplement: Supplementary Information — Supplementary Figures 1 - 5, Supplementary Tables 1 - 4, Supplementary Note 1 and Supplementary References [file ncomms11812-s1.pdf]

Martínez-Martínez et al., **Supplementary Figure 1**

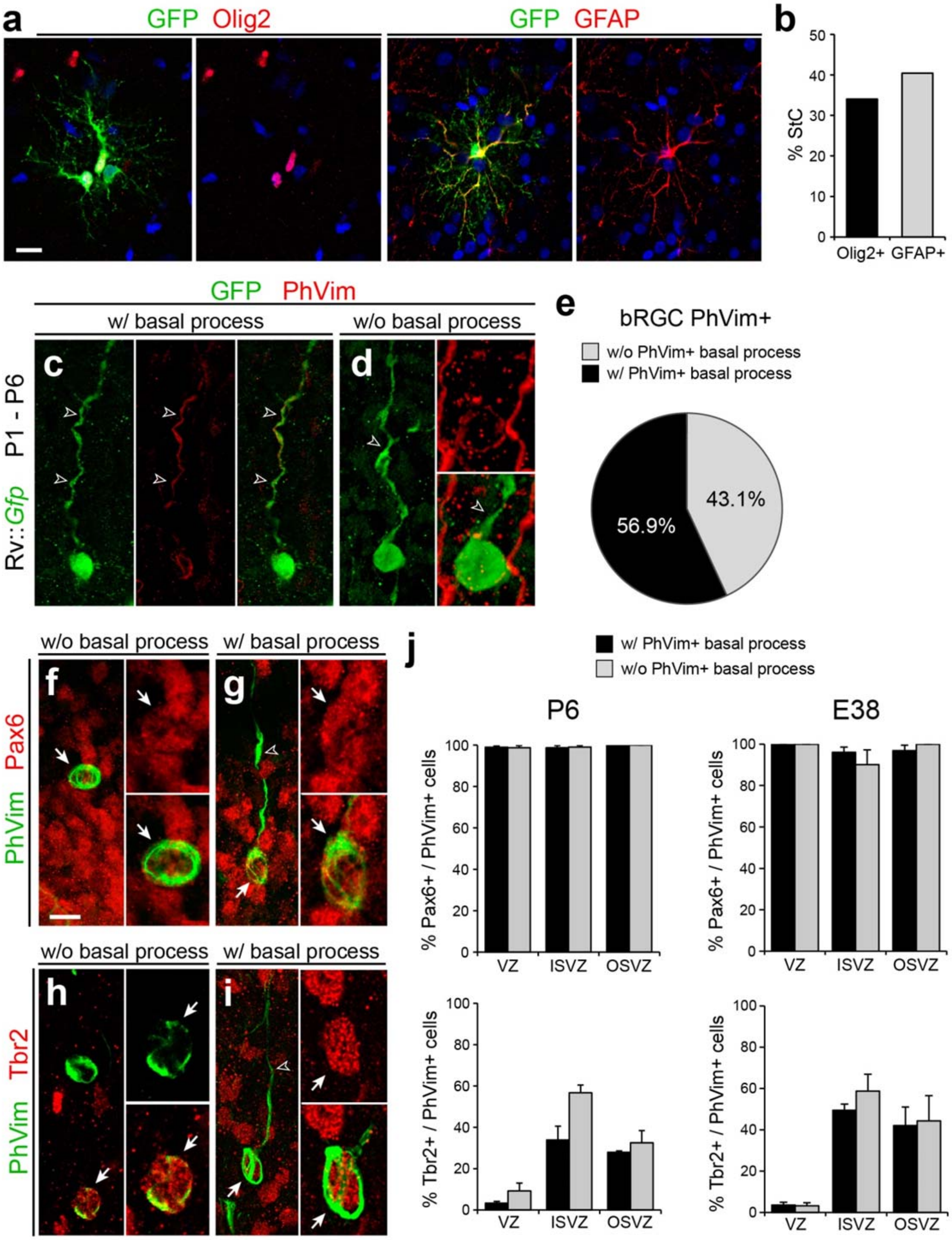

Supplementary Figure 1. Marker expression analysis of aRGC, bRGC and StC cells. (**a,b**) Examples of StC cells at P14 expressing Olig2 or GFAP, and quantification (*n* = 47 cells, Olig2; 47 cells, GFAP; 2 kits per group). (**c-e**) GFP+ bRGCs in ISVZ at P6 labeled after ventricular injection of rv::*Gfp* at P1, displaying PhVim label without or with a basal process, and relative abundance (*n* = 54 cells, 4 animals; mean value). (**f-j**) PhVim+ cells in VZ, ISVZ and OSVZ at P6 and E38, without or with a basal process, expressing Pax6 or Tbr2, and relative abundance (**j**) (P6: Pax6, *n* = 788 cells in VZ, 692 cells in ISVZ, 292 cells in OSVZ, 3 animals; Tbr2, *n* = 514 cells in VZ, 1,012 cells in ISVZ, 526 cells in OSVZ, 3 animals. E38: Pax6, *n* = 126 cells in VZ, 215 cells in ISVZ, 118 cells in OSVZ, 3 animals; Tbr2, *n* = 456 cells in VZ, 420 cells in ISVZ, 237 cells in OSVZ, 3 animals; mean values + s.e.m.). Detail images demonstrating co-localization are single confocal planes. Scale bars, 10 μm.

Martínez-Martínez et al., **Supplementary Figure 2**

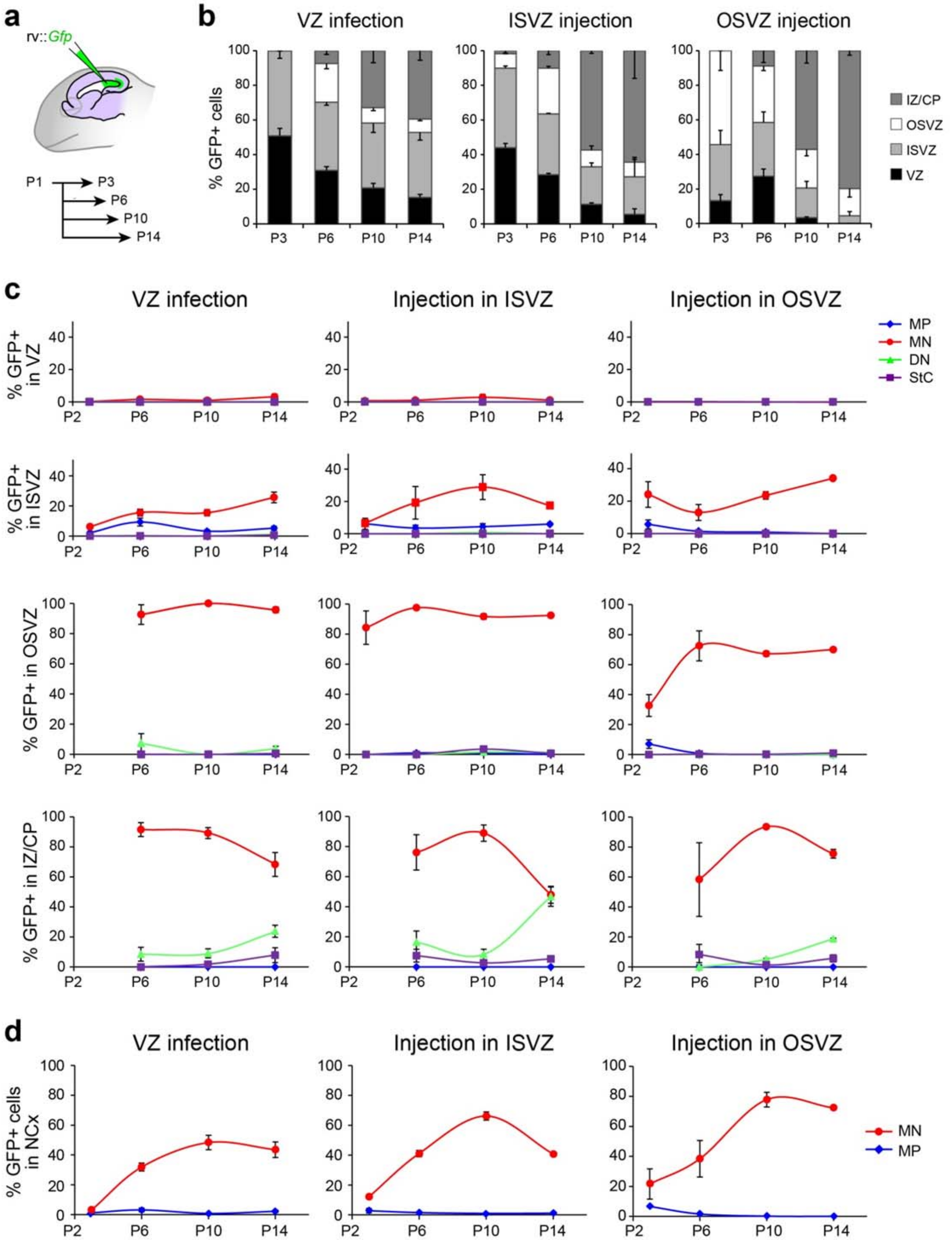

Supplementary Figure 2. Laminar distribution and types of cells in postnatal lineages. (**a,b**) Laminar distribution of GFP+ cells after injection of rv::*Gfp* in VZ (lateral ventricle), ISVZ or OSVZ at P1, and analysis at the indicated postnatal ages. (**c**) Relative abundance of non-RGC GFP+ cells within the indicated cortical layers bearing the identified morphologies at the indicated postnatal ages after injection of rv::*Gfp* at P1 in VZ (lateral ventricle), in ISVZ or in OSVZ. (**d**) Relative abundance of GFP+ MN and MP cells in the neocortex at the indicated postnatal ages after injection of rv::*Gfp* at P1 in VZ, ISVZ or OSVZ. Data are mean values ± s.e.m. Number of cells and animals analyzed is: infection of VZ, *n* = 1,094 cells, 4 animals, P3; 5,544 cells, 4 animals, P6; 1,499 cells, 5 animals, P10; 4,196 cells, 3 animals, P14. Injection in ISVZ, *n* = 962 cells, 2 animals, P3; 1,063 cells, 2 animals, P6; 2,705 cells, 3 animals, P10; 3,107 cells, 2 animals, P14. Injection in OSVZ: *n* = 420 cells, 2 animals, P3; 1,092 cells, 3 animals, P6; 3,742 cells, 3 animals, P10; 1,663 cells, 2 animals, P14. IZ, intermediate zone; CP, cortical plate.

Martínez-Martínez et al., **Supplementary Figure 3**

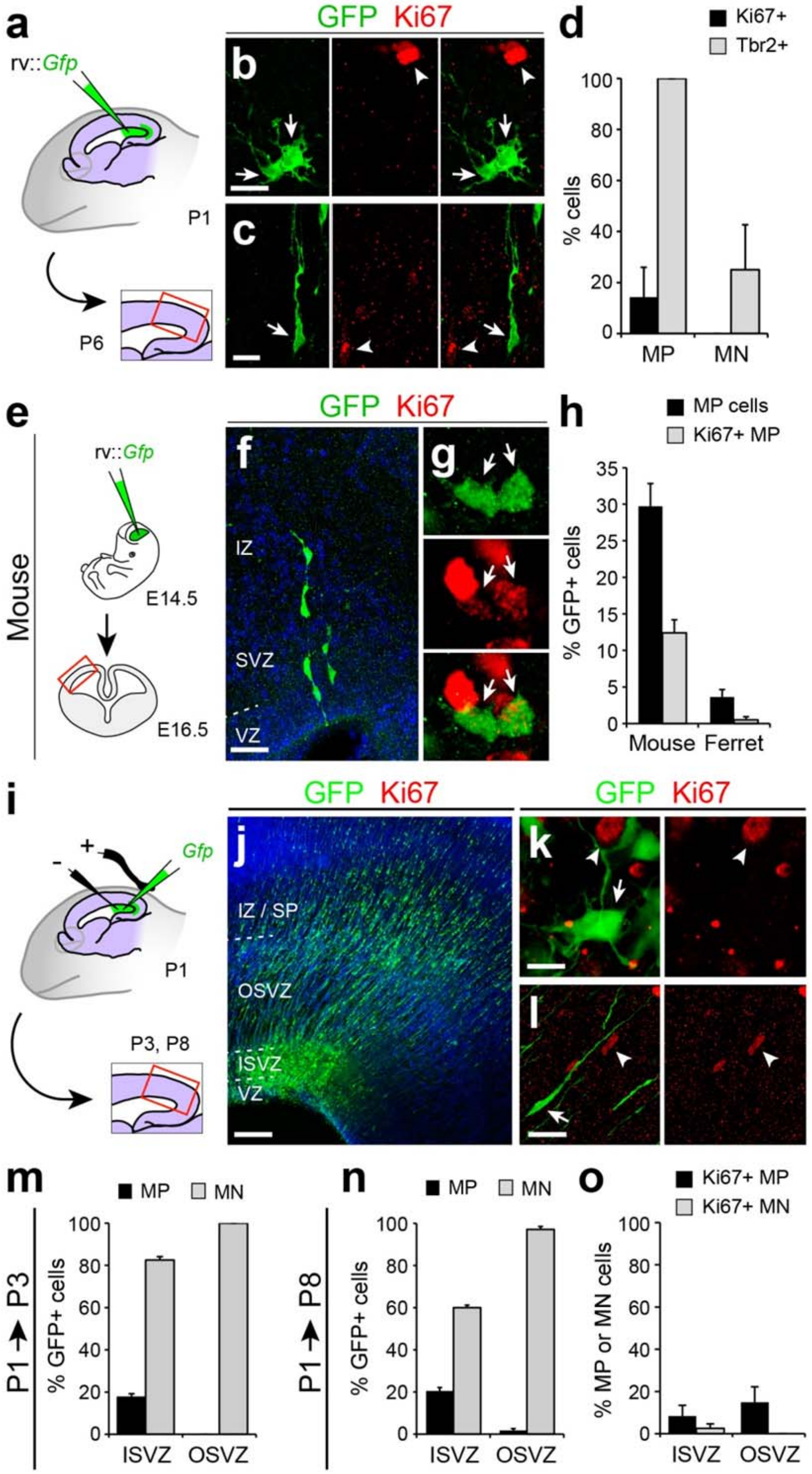

Supplementary Figure 3. Intermediate Progenitor Cells are scarce in the gyrated ferret cortex. (**a-d**) GFP-labeling of MP and MN cells after intraventricular delivery of rv::*Gfp* at P1 and analysis at P6. All MP cells were positive for Tbr2 but few for Ki67 (**b,d**; *n* = 15 cells, 3 animals, Ki67; 17 cells, 3 animals, Tbr2), while few MN cells were Tbr2+ and none were Ki67+ (**c,d**; *n* = 38 cells, 3 animals, Ki67; 40 cells, 3 animals, Tbr2; mean values + s.e.m.). (**e-h**) GFP-labeling of mouse MP and MN cells after intraventricular delivery of rv::*Gfp in utero* at E14.5 and analysis at E16.5, stages of cortical development equivalent to P1-P6 in ferret. Arrows in (**g**) indicate Ki67+ MP cells. (**h**) The abundance of MP cells (total) and Ki67+ MP cells in mouse was much higher than in ferret (Mouse: MP, *n* = 338 cells, 7 embryos; Ki67+ MP, *n* = 76 cells, 7 embryos. Ferret: MP, *n* = 5,544 cells, 4 animals; Ki67+ MP, *n* = 15 cells, 3 animals). (**i-o**) Ferrets were electroporated with *Gfp*-encoding DNA at P1 and analyzed at P3 or P8. (**k,l**) Examples of MN (**k**) and MP (**l**) cells at P8 negative for Ki67. (**m,n**) Abundance of GFP+ MP and MN cells at P3 and P8, showing that very few GFP+ cells in ISVZ and OSVZ were MP at both stages, although their frequency was higher after electroporation than after rv::*Gfp* infection (compare to **h**) (P3: *n* = 1,749 cells, ISVZ; 39 cells, OSVZ; 3 animals per group. P8: *n* = 2,314 cells, ISVZ; 3,799 cells, OSVZ; 4 animals per group). (**o**) Quantification of GFP+ MP and MN cells within ISVZ and OSVZ positive for Ki67, demonstrating that very few MP and nearly none of the MN cells were Ki67+ (ISVZ, *n* = 172 cells, MP; 112 cells, MN; OSVZ, *n* = 38 cells, MP; 282 cells, MN; 3 animals per group). Scale bars in **b,c**, 10 μm; in **f**, 30 μm; in **j**, 150 μm; in **k**, 5 μm; in **l**, 20 μm.

Martínez-Martínez et al., **Supplementary Figure 4**

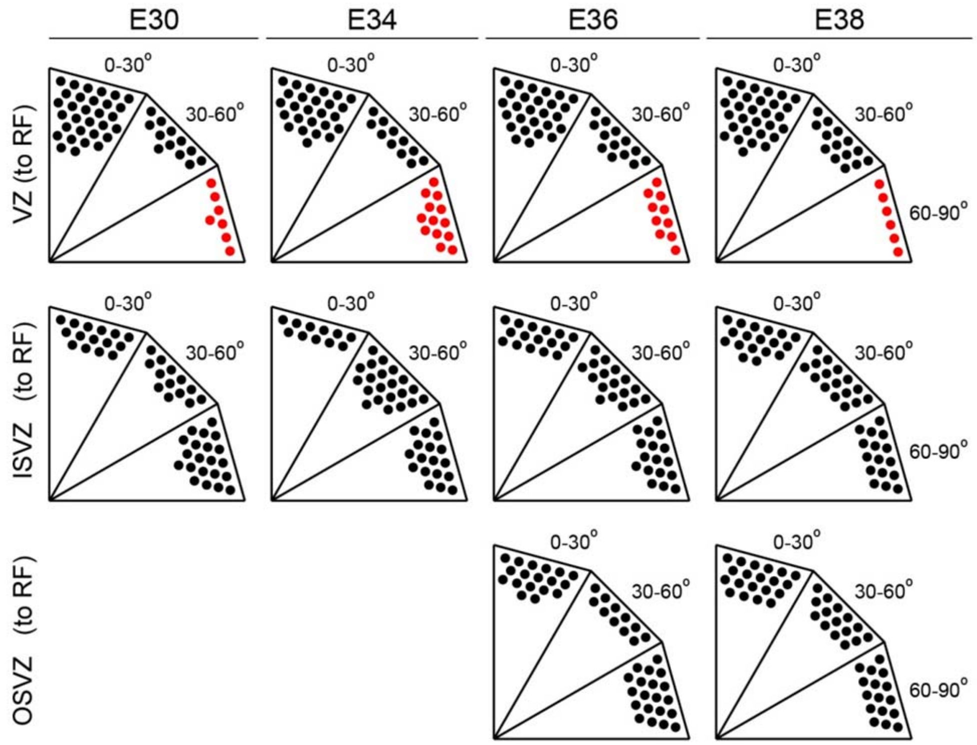

Supplementary Figure 4. Transient increase of horizontal mitotic cleavage planes in VZ progenitors at E34. Distribution of orientations for apical mitoses in VZ with respect to the Radial Fiber scaffold (RF), and for basal mitoses in ISVZ and OSVZ with respect to RF, across embryonic stages. Each dot represents 2% of mitoses (VZ: *n* = 91 cells, 8 embryos, E30; 90 cells, 2 embryos, E34; 124 cells, 2 embryos, E36; 69 cells, 3 embryos, E38. ISVZ: *n* = 128 cells, 8 embryos, E30; 40 cells, 2 embryos, E34; 96 cells, 2 embryos, E36; 81 cells, 3 embryos, E38. OSVZ: *n* = 49 cells, 2 embryos, E36; 61 cells, 3 embryos, E38). Horizontal cleavage planes in VZ mitoses doubled in abundance transiently at E34, coincident with the onset of bRGC peak production.

Martínez-Martínez et al., **Supplementary Figure 5**

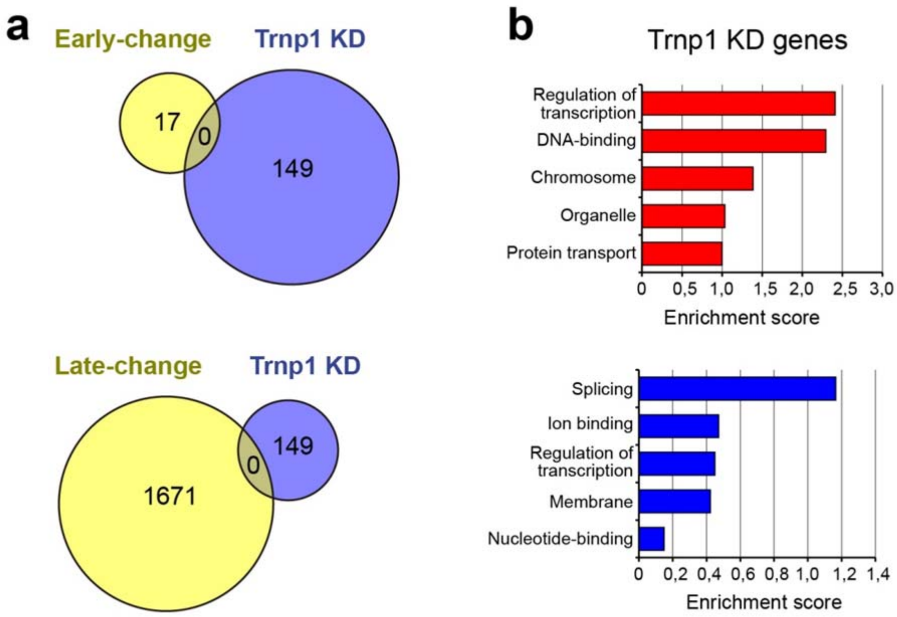

Supplementary Figure 5. Comparison of differentially-expressed gene sets and functional gene annotation between Trnp1 knock-down microarray data (Trnp1 KD) from ref. [1] and microarray data from this study. (a) None of the "early-change" genes or "late-change" genes identified in this study was reported as differentially expressed upon Trnp1 knock-down in mouse cortex. (b) Top five clusters of functional gene annotation terms with the highest enrichment scores for up- (red) and down-regulated (blue) genes upon Trnp1 knock-down.

Supplementary Table 1 - Number of cells and animals analyzed for each survival period in the quantification of cell lineages shown in Figure 4.

Layer of rv::*Gfp* delivery	Age	P1-P3	P1-P6	P1-P10	P1-P14
VZ	N cells	1,094	5,544	1,499	4,196
	N kits	4	4	5	3
ISVZ	N cells	962	1,063	2,705	3,107
	N kits	2	2	3	2
OSVZ	N cells	420	1,092	3,742	1,663
	N kits	2	3	3	2

Supplementary Table 2 - List of genes with “Early” change profile.

Gene Name	ProbeID	Fold Change	adj.P.Value
LYPD1	CUST_10832_PI427051300	**4.30**	**1.51E-02**
LYPD1	CUST_10833_PI427051300	**4.29**	**2.15E-02**
SLITRK2	CUST_34563_PI427051300	**3.77**	**1.32E-02**
SLITRK2	CUST_43074_PI427051300	**3.53**	**1.32E-02**
SLITRK2	CUST_34561_PI427051300	**3.49**	**2.73E-02**
SLITRK2	CUST_34560_PI427051300	**3.46**	**1.40E-02**
FAM167A	CUST_6407_PI427051300	**2.36**	**1.51E-02**
VCAM1	CUST_36846_PI427051300	**2.08**	**1.32E-02**
LOC100127983	CUST_10110_PI427051300	**-2.05**	**4.85E-02**
FRZB	CUST_7108_PI427051300	**-2.13**	**4.62E-02**
DCN	CUST_25383_PI427051300	**-2.19**	**2.23E-02**
WLS	CUST_21015_PI427051300	**-2.33**	**1.57E-02**
AHR	CUST_22367_PI427051300	**-2.33**	**1.51E-02**
ZFPM2	CUST_37486_PI427051300	**-2.43**	**1.51E-02**
SUSD1	CUST_18556_PI427051300	**-2.44**	**2.23E-02**
INA	CUST_8983_PI427051300	**-2.46**	**2.86E-02**
EDN3	CUST_5627_PI427051300	**-2.60**	**1.32E-02**
SLIT1	CUST_17682_PI427051300	**-2.79**	**2.49E-02**
EFNA5	CUST_26045_PI427051300	**-2.95**	**1.77E-02**
PTPRE	CUST_32592_PI427051300	**-3.62**	**5.86E-03**
COL21A1	CUST_4155_PI427051300	**-3.74**	**2.41E-02**

List of genes with “Early” change profile in ferret VZ (different between E30 and E34, but not between E34 and P1), indicating microarray probe identification, value of fold-change (E34 to E30) and adjusted *p* value.

Supplementary Table 3 - List of genes with “Late” change profile.

Gene Name	ProbeID	Fold Change	adj.P.Value
APOE	CUST_989_PI427051300	17.85	2.78E-06
APOE	CUST_988_PI427051300	16.05	1.42E-06
CTSW	CUST_4601_PI427051300	15.08	3.07E-03
GJB6	CUST_7477_PI427051300	13.08	1.69E-06
SLC7A11	CUST_17633_PI427051300	12.02	2.98E-04
ZCCHC24	CUST_21239_PI427051300	11.42	1.80E-04
ZCCHC24	CUST_21240_PI427051300	11.06	5.21E-05
LOC480072	CUST_41591_PI427051300	9.73	9.11E-05
TRIL	CUST_19952_PI427051300	9.51	2.79E-05
KLF9	CUST_28985_PI427051300	9.48	4.82E-05
BATF2	CUST_1620_PI427051300	9.43	8.91E-05
BBOX1	CUST_1626_PI427051300	9.31	5.17E-04
PID1	CUST_13954_PI427051300	9.26	1.80E-04
TRIL	CUST_19950_PI427051300	9.07	6.56E-05
MGLL	CUST_11413_PI427051300	8.86	4.82E-05
OLIG1	CUST_13076_PI427051300	8.79	6.39E-04
AQP4	CUST_22747_PI427051300	8.74	2.63E-03
GJB6	CUST_7478_PI427051300	8.61	1.42E-06
LTBP1	CUST_29561_PI427051300	8.48	6.78E-03
TIMP3	CUST_19212_PI427051300	8.46	3.68E-05
ZDHHC17	CUST_37412_PI427051300	8.15	5.56E-03
RFTN2	CUST_33105_PI427051300	7.99	1.65E-03
GHRL	CUST_7445_PI427051300	7.92	2.10E-03
C21orf63	CUST_2380_PI427051300	7.71	7.82E-05
MIER1	CUST_30123_PI427051300	7.65	3.10E-03
SCRG1	CUST_16613_PI427051300	7.61	5.24E-05
SMOX	CUST_34688_PI427051300	7.39	5.43E-03
LTBP1	CUST_29563_PI427051300	7.24	2.00E-03
NR1D1	CUST_12809_PI427051300	7.10	2.64E-03
FAM19A3	CUST_6464_PI427051300	6.87	8.97E-04
**TRNP1**	**CUST_20041_PI427051300**	2.86	2.44E-04
GTPBP8	CUST_8086_PI427051300	-5.25	1.68E-03
CSRP2	CUST_4506_PI427051300	-5.27	8.98E-04
CSRP2	CUST_4509_PI427051300	-5.27	2.40E-03
ATP11A	CUST_23113_PI427051300	-5.32	1.44E-02
HIF1AN	CUST_8349_PI427051300	-5.36	1.55E-02
HBD	CUST_27715_PI427051300	-5.37	7.60E-03
HIF1AN	CUST_8350_PI427051300	-5.38	1.78E-02
LRTOMT	CUST_10747_PI427051300	-5.40	1.40E-02
PRR13	CUST_14866_PI427051300	-5.41	9.19E-03
RBM10	CUST_32930_PI427051300	-5.41	1.76E-03

KCTD21	CUST_9436_PI427051300	**-5.48**	**4.52E-03**
DCC	CUST_4889_PI427051300	**-5.56**	**3.26E-04**
CXCR7	CUST_4677_PI427051300	**-5.58**	**1.58E-03**
HBM	CUST_8197_PI427051300	**-5.58**	**5.64E-03**
C14orf101	CUST_23694_PI427051300	**-5.65**	**3.08E-03**
FKBP4	CUST_6948_PI427051300	**-5.68**	**3.92E-03**
GRB10	CUST_7910_PI427051300	**-5.86**	**1.36E-03**
MTCH2	CUST_30311_PI427051300	**-5.90**	**3.09E-03**
NARS2	CUST_12260_PI427051300	**-5.99**	**2.29E-03**
JPH3	CUST_9259_PI427051300	**-6.39**	**5.91E-03**
HBA1	CUST_8191_PI427051300	**-6.63**	**4.64E-04**
MTCH2	CUST_30310_PI427051300	**-6.69**	**3.94E-03**
MTCH2	CUST_30309_PI427051300	**-6.76**	**5.06E-03**
NEUROD6	CUST_12506_PI427051300	**-7.34**	**3.67E-04**
HBA1	CUST_8192_PI427051300	**-7.42**	**1.04E-03**
HBM	CUST_8196_PI427051300	**-7.45**	**1.55E-03**
OBFC2A	CUST_31172_PI427051300	**-8.20**	**5.59E-04**
C3orf63	CUST_23857_PI427051300	**-8.32**	**8.88E-04**
NDNF	CUST_12338_PI427051300	**-9.73**	**1.69E-06**
CXCR7	CUST_4678_PI427051300	**-16.11**	**1.11E-05**

List of genes with “Late” change profile in ferret VZ (different between E34 and P1, but not between E30 and E34) indicating microarray probe identification number, value of fold-change (P1 compared to E34) and adjusted *p* value. Only the top 30 probes with highest positive fold-change, 30 with highest negative, plus TRNP1, are included.

Supplementary Table 4 - Number of cells and animals analyzed for each survival period in the quantification of cell lineages shown in Figures 6 and 7.

	Age	Treatment	N cells	N embryos
Trnp1 overexpression	E34-E36	Control	931	8
		Trnp1	302	7
Trnp1 blockade	P1-P3	Control	1,094	4
		DN-Trnp1	731	2
Trnp1 overexpression	E34-P0	Control	613	3
		Trnp1	211	4
Cdh1 blockade	E30-E32	Control	866	2
		DN-Cdh1	1,001	2
Cdh1 overexpression	E34-E36	Control	1,111	3
		Cdh1	954	2

Supplementary Note

PhVim labeling does not faithfully recapitulate progenitor cell morphology

Our marker expression analyses demonstrated that the vast majority of GFP+ cells morphologically identified as aRGCs and bRGCs were positive for Ki67 and Pax6, and in addition that 23.7% of aRGCs and 34.3% of bRGCs also expressed Tbr2 (**Fig. 1**). This was surprising because in mouse Tbr2 is a marker of IPCs but not RGCs[2-4], also reported previously in ferret and human[5-8]. However, these observations were fully consistent with more recent analyses in macaque embryos [9]. To confirm these findings we performed anti-phosphovimentin (PhVim) stains. PhVim is commonly used to identify the morphology of progenitor cells at mitosis, thus to distinguish progenitor cells with a basal process (putative RGCs) from those without one (putative IPCs)[4-6,10-14]. First we investigated the pattern of PhVim labeling in morphologically-identified GFP+ bRGCs. Unexpectedly, we found that of all GFP+ bRGCs positive for PhVim (in mitosis), only 56.9% had a basal process, whereas 43.1% lacked a PhVim+ basal process (**Supplementary Fig. 1c-e**). To reconcile these results with our above marker expression analyses, we studied the expression of Pax6 and Tbr2 in PhVim+ cells with and without a basal process. At two different developmental stages we found nearly identical results, and very similar to our above analyses using rv::*Gfp*: Pax6 was expressed by 96.0-100% of cells with a PhVim+ basal process, but then also by 90-100% of cells without a PhVim+ basal process (**Supplementary Fig. 1f,g,j**). Similarly, Tbr2 was expressed at similar frequencies by cells with and without a PhVim+ basal process in all three germinal layers (**Supplementary Fig. 1h-j**). This was contrary to mouse, where bRGCs are Pax6+/Tbr2-, and IPCs are mostly Pax6-/Tbr2+ [2,4,15]. These results demonstrated that PhVim labeling patterns do not recapitulate progenitor cell morphology, and also that bRGCs in gyrencephalic species are molecularly diverse, varying in Tbr2 expression and PhVim pattern. In addition, the frequent expression of Tbr2 in bRGCs suggested that these might be the main neurogenic progenitors in the ferret cortex as recently shown in macaque embryos, where IPCs are very scarce as we found in ferret[9].

IPCs are very scarce in ferret

Our cell lineage tracing experiments in early postnatal ferrets labeled a variety of cell types across cortical layers that included, in addition to aRGCs and bRGCs, cells with multipolar morphology (MP), bipolar cells resembling migrating neurons (MN), differentiating neurons with a branched apical dendrite (DN) and cells with a star-like morphology (StC), the latter including cells in the astrocyte and oligodendrocyte lineages (**Fig. 1, Supplementary Fig. 1a,b**). All these cell types were revealed by GFP regardless of the layer of rv::*Gfp* delivery, progressively accumulated in IZ and CP at late stages (**Supplementary Fig. 2a,b**), and systematically predominated in the OSVZ and IZ/CP, particularly MNs and DNs (**Supplementary Fig. 2c**). As expected, DNs were only found in the upper CP and increased in abundance at later stages (P14; **Supplementary Fig. 2c**).

MP cells had the typical morphology of IPCs [16,17], but their relative abundance was very low (<7% of GFP+ cells; **Supplementary Fig. 2c,d**), especially considering that many cells in GFP+ clones eventually differentiated as neurons (**Supplementary Fig. 2c**). Moreover, SVZ cells with multipolar morphology include IPCs and newborn neurons [18]. To specifically identify IPCs we stained against Ki67 (marker of cycling cells) and Tbr2 (marker of IPCs and newborn neurons). This analysis was performed at P6 after rv::*Gfp* injection in VZ at P1, to obtain the largest number of GFP+ MP cells (**Supplementary Fig. 2**, **3a**). Whereas 100% of MPs were Tbr2+, only 14.3% were Ki67+, indicating that only a minority of the already few MPs were IPCs (**Supplementary Fig. 3b,d**). In contrast to the unexpected scarcity of MP cells, MN cells were very abundant (**Supplementary Fig. 2**), so we analyzed if these could be IPCs with bipolar morphology. Only 25.0% of MNs were Tbr2+ and none

were Ki67+ (**Supplementary Fig. 3c,d**), consistent with these cells not being IPCs but rather newborn cortical neurons.

The above results using rv::*Gfp* seemed to indicate that IPCs were extremely rare in ferret, contrary to mouse[2,3,19]. An alternative was that our pleiotropic retroviral vectors might somehow have a different cellular specificity between mouse and ferret. To discard this possibility we injected rv::*Gfp* in mouse embryos at E14.5 and analyzed at E16.5, a period of cortical development equivalent to P1-P6 in ferret (**Supplementary Fig. 3e**). MP cells represented 29.7% of all GFP+ cells in mouse, eight times more than in ferret (3.6%; **Supplementary Fig. 3f-h**). Ki67 stains revealed that in mouse embryos 12.4% of all GFP+ cells were putative IPCs (Ki67+ MP cells; **Supplementary Fig. 3g**), while these represented only 0.5% in ferret (**Supplementary Fig. 3h**). This further supported that IPCs may be very scarce in ferret, representing very few of the already small population of MP cells. To further confirm the unexpected scarcity of IPCs in ferret we electroporated *Gfp*-encoding plasmids in VZ of newborn kits, because this method labels abundant pyramidal neurons in the juvenile ferret cortex [20] and so it must label their cellular lineage earlier in development (**Supplementary Fig. 3i,j**). In contrast to retroviral infection, electroporation labeled a much greater proportion of cells with MP and MN morphology along the same developmental period (**Supplementary Fig. 3j-n**). However, the proportion of MP and MN cells positive for Ki67 was again extremely low or null (**Supplementary Fig. 3o**), demonstrating that their vast majority was non-proliferative and, thus, that IPCs are extremely scarce in the early postnatal ferret.

Taken together, our analyses demonstrated that RGCs (apical and basal) represented the vast majority of progenitor cells in the developing ferret cerebral cortex, and that while nearly all expressed Pax6, they were heterogeneous in Tbr2 expression and in the pattern of PhVim stain, where presence or absence of a PhVim+ basal process did not recapitulate actual progenitor cell morphology at mitosis. Importantly, our analyses demonstrated that non-RGC progenitors are extremely infrequent in ferret compared to RGCs. Therefore, ferret cortical neurogenesis does not rely on multipolar IPCs, as in rodents[2,16,17], but RGCs seem to be the main neurogenic progenitors, thus departing from rodents and resembling macaques, where IPCs are also very scarce and bRGCs are highly neurogenic[2,9,16,17].

Comparison between DEGS across the critical period and upon Trnp1 knock-down

Our microarray gene expression analysis revealed the existence of two sets of differentially-expressed genes (DEGs): "Early-change" genes, whose expression levels changed significantly only between E30 and E34; and "Late-change" genes, whose expression changed only between E34 and P1 (**Fig. 5**). This distinguished those genes that changed early with the increase in bRGC generation, and those that changed late with the decrease of bRGC generation. Among the "late" genes we found *Trnp1*, a gene encoding for a DNA-associated protein which has been suggested to regulate the expression or function of other genes, and previously shown to block bRGC production in the embryonic mouse cortex[1]. We compared our lists of early and late DEGs with the list of genes differentially-expressed upon experimental knock-down of Trnp1 in mouse embryos, obtained from ref. [1]. None of the 17 "early" or 1,671 "late" genes was present among the 149 DEGs identified upon Trnp1 knock-down (**Supplementary Fig. 5a**). Because similar biological functions may be performed by redundant genes, we next compared the functional gene annotation term clusters between these three sets of DEGs (**Supplementary Fig. 5b**). With this analysis we found some coherent similarities, such as with the term "*organelle*": top 3 cluster in down-regulated late genes and top 4 cluster in up-regulated Trnp1-KD genes; and also the related terms "*ribonucleotide binding*" and "*regulation of transcription*", top 2 cluster in down-regulated late genes and top 1 cluster in up-regulated Trnp1-KD genes, respectively.

References

1. Stahl R*, et al.* Trnp1 regulates expansion and folding of the mammalian cerebral cortex by control of radial glial fate. *Cell* **153**, 535-549 (2013).

2. Kowalczyk T*, et al.* Intermediate neuronal progenitors (basal progenitors) produce pyramidal-projection neurons for all layers of cerebral cortex. *Cereb Cortex* **19**, 2439-2450 (2009).

3. Englund C*, et al.* Pax6, Tbr2, and Tbr1 are expressed sequentially by radial glia, intermediate progenitor cells, and postmitotic neurons in developing neocortex. *J Neurosci* **25**, 247-251 (2005).

4. Wang X, Tsai JW, Lamonica B, Kriegstein AR. A new subtype of progenitor cell in the mouse embryonic neocortex. *Nat Neurosci* **14**, 555-561 (2011).

5. Fietz SA*, et al.* OSVZ progenitors of human and ferret neocortex are epithelial-like and expand by integrin signaling. *Nat Neurosci* **13**, 690-699 (2010).

6. Hansen DV, Lui JH, Parker PR, Kriegstein AR. Neurogenic radial glia in the outer subventricular zone of human neocortex. *Nature* **464**, 554-561 (2010).

7. Reillo I, de Juan Romero C, Garcia-Cabezas MA, Borrell V. A role for intermediate radial glia in the tangential expansion of the Mammalian cerebral cortex. *Cereb Cortex* **21**, 1674-1694 (2011).

8. Gertz CC, Lui JH, LaMonica BE, Wang X, Kriegstein AR. Diverse behaviors of outer radial glia in developing ferret and human cortex. *J Neurosci* **34**, 2559-2570 (2014).

9. Betizeau M*, et al.* Precursor diversity and complexity of lineage relationships in the outer subventricular zone of the primate. *Neuron* **80**, 442-457 (2013).

10. Kelava I*, et al.* Abundant Occurrence of Basal Radial Glia in the Subventricular Zone of Embryonic Neocortex of a Lissencephalic Primate, the Common Marmoset Callithrix jacchus. *Cereb Cortex* **22**, 469-481 (2012).

11. Garcia-Moreno F, Vasistha NA, Trevia N, Bourne JA, Molnar Z. Compartmentalization of cerebral cortical germinal zones in a lissencephalic primate and gyrencephalic rodent. *Cereb Cortex* **22**, 482-492 (2012).

12. Martinez-Cerdeno V*, et al.* Comparative analysis of the subventricular zone in rat, ferret and macaque: evidence for an outer subventricular zone in rodents. *PLoS One* **7**, e30178 (2012).

13. Reillo I, Borrell V. Germinal zones in the developing cerebral cortex of ferret: ontogeny, cell cycle kinetics, and diversity of progenitors. *Cereb Cortex* **22**, 2039-2054 (2012).

14. Weissman T, Noctor SC, Clinton BK, Honig LS, Kriegstein AR. Neurogenic radial glial cells in reptile, rodent and human: from mitosis to migration. *Cereb Cortex* **13**, 550-559 (2003).

15. Arai Y*, et al.* Neural stem and progenitor cells shorten S-phase on commitment to neuron production. *Nat Commun* **2**, 154 (2011).

16. Miyata T, Kawaguchi A, Saito K, Kawano M, Muto T, Ogawa M. Asymmetric production of surface-dividing and non-surface-dividing cortical progenitor cells. *Development* **131**, 3133-3145 (2004).

17. Noctor SC, Martinez-Cerdeno V, Ivic L, Kriegstein AR. Cortical neurons arise in symmetric and asymmetric division zones and migrate through specific phases. *Nat Neurosci* **7**, 136-144 (2004).

18. Tabata H, Nakajima K. Multipolar migration: the third mode of radial neuronal migration in the developing cerebral cortex. *J Neurosci* **23**, 9996-10001 (2003).

19. Borrell V*, et al.* Slit/Robo signaling modulates the proliferation of central nervous system progenitors. *Neuron* **76**, 338-352 (2012).

20. Borrell V. In vivo gene delivery to the postnatal ferret cerebral cortex by DNA electroporation. *J Neurosci Methods* **186**, 186-195 (2010).
